# Outer-surface charge modulation of photothermal diffusion voltage enables ultrasensitive sensing in nanofluidic membranes

**DOI:** 10.1039/d5sc10110g

**Published:** 2026-02-25

**Authors:** Yihan Ma, Xinyi Yang, Bingquan Qi, Xiaoping Yang, Zhengxu He, Ning Feng, Li Dai, Aiqing Zhang, Yu Huang, Fan Xia

**Affiliations:** a Key Laboratory of Catalysis and Energy Materials Chemistry of Ministry of Education, Hubei Key Laboratory of Catalysis and Materials Science, South-Central Minzu University Wuhan 430074 China; b State Key Laboratory of Geomicrobiology and Environmental Changes, Engineering Research Center of Nano-Geomaterials of Ministry of Education, Faculty of Materials Science and Chemistry, China University of Geosciences Wuhan 430074 China yuhuang@cug.edu.cn

## Abstract

Precise ionic transport regulation is central to nanofluidic sensing, yet quantitative readout at ultratrace analyte levels remains challenging because conventional externally biased measurements primarily transduce target binding through resistance changes. When trace analytes induce negligible steric variation, the system resistance is essentially unchanged, yielding insufficient ionic current contrast. Here we develop an outer-surface charge-modulated, photothermal diffusion voltage-driven strategy in an MXene nanofluidic membrane. Under 808 nm illumination, the strong photothermal conversion of Ti_3_C_2_T_*x*_ establishes a stable transmembrane temperature gradient across K^+^-permselective lamellar nanochannels, generating a tunable photothermal diffusion voltage (*V*_diff_). Trace-level binding events markedly modulate the outer-surface charge density, thereby altering the K^+^ transference number and amplifying minute charge variations into pronounced changes in *V*_diff_ and the zero-bias ionic current, even when steric hindrance and resistance remain nearly constant. Using microcystin-LR (MC-LR) as a model toxin, this strategy enables ultratrace detection down to 1 × 10^−7^ µg L^−1^, delivering a 10^5^-fold sensitivity enhancement over conventional external voltage-driven readout while retaining high selectivity against structural analogues, and reliable quantification in real water matrices. This work establishes a light-addressable route to actively regulate nanofluidic voltages *via* outer-surface charge, opening opportunities for photoresponsive nanofluidic sensors and iontronic circuitry.

## Abstract 2

A photothermal diffusion voltage-driven strategy in MXene nanofluidic membranes amplifies outer-surface charge modulation, achieving ultrasensitive sensing with a 10^5^-fold enhancement over conventional methods.

## Graphical abstract

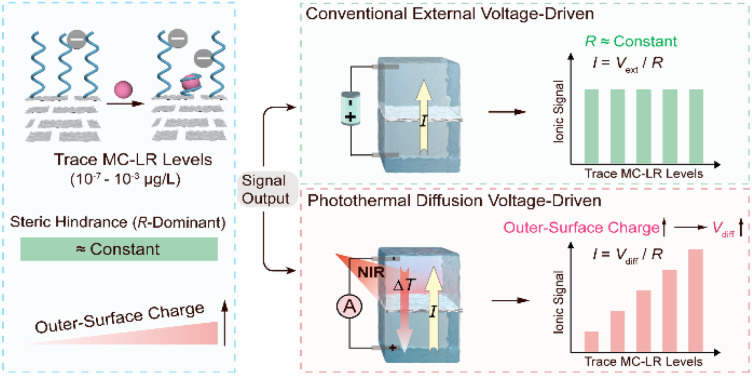

## Introduction

In biological systems, protein nanopores precisely regulate transmembrane ionic currents, enabling selective transport of molecules and maintaining essential physiological functions such as nerve signal transmission, muscle contraction, and osmotic homeostasis.^1–3^ These natural channels represent highly optimized systems that couple structural dynamics with functional specificity, utilizing subtle variations in charge distribution, steric hindrance, and membrane composition to achieve selective and efficient ion transport.^4–6^ The remarkable performance of biological nanopores has inspired extensive efforts to develop artificial solid-state nanochannels that mimic their selective transport behavior.^7–10^ Using conventional external voltage-driven sensing strategies, target-specific recognition probes are integrated onto the surfaces of solid-state nanochannels so that molecular binding events are transduced into measurable ionic current changes through alterations in interfacial properties such as wettability, steric hindrance, and surface charge.^11–14^ This biomimetic approach has enabled the detection of a wide range of targets, from small ions and biomolecules to pathogens,^15–18^ as demonstrated by advances in electrochemical aptamer-based biosensors,^19,20^ responsive nanochannels,^21,22^ and bio-inspired smart nanochannels.^23,24^ In these systems, ionic currents are typically recorded at a fixed external voltage (*V*_ext_) and therefore follow Ohm's law (*I* = *V*/*R*), so the sensing signal mainly arises from target-induced changes in the effective channel resistance *R*. However, at ultratrace analyte concentrations, the variations in wettability, steric hindrance, and surface charge induced within the nanochannels are extremely subtle, leading to negligible changes in *R* and insufficient ionic-current contrast. As a result, the detection sensitivity of current solid-state nanochannel sensors still lags far behind that of biological protein nanopores, which can reach single-molecule precision.^25–27^ To enhance these modest resistance changes within the fixed-voltage framework, most external voltage-driven nanochannel sensors exploit steric hindrance, where target binding triggers conformational changes in recognition probes that slightly alter the effective pore size and thus modulate ionic transport.^28–30^ A variety of signal-amplification schemes, such as super-sandwich assays,^31,32^ rolling circle amplification,^33,34^ and cyclic detection strategies,^35,36^ have been integrated to magnify this steric effect. Nevertheless, the extent of steric modulation is intrinsically limited by the structures of recognition probes, many of which cannot be readily engineered to generate sufficiently large geometric changes, thereby constraining further improvements in sensitivity and generality within the conventional external voltage-driven strategy.

This situation motivates re-examination of biological protein nanopores, where many systems, such as the bacterial porin OmpF, possess charged vestibules that act as electrostatic gates to preconcentrate counter-ions or repel co-ions, thereby strongly influencing ionic transport.^37,38^ These observations suggest that modulation of surface charge, rather than steric hindrance alone, plays a decisive role in regulating ion transport and provides a promising direction for constructing highly sensitive, universal nanochannel sensors. Inspired by the structures of biological protein nanopores, we have previously demonstrated that selective functionalization of the outer surface alone is sufficient to modulate transmembrane ion transport, achieved by precisely constructing nanochannels with spatially resolved inner and outer surface chemistries.^39^ Compared with the geometrically confined inner surface, the outer surface lies in an open and accessible environment, enabling efficient probe immobilization, direct physicochemical characterization, and highly effective interaction with target analytes.^40–42^ Subsequent studies further established that tuning outer-surface charge provides a powerful means of regulating ion transport and amplifying sensing responsiveness in nanochannel systems.^43–50^ Building on this concept, we recently introduced partitioned-interface nanochannels, revealing that variations in outer-surface charge density dominate ion transport behavior, outweighing steric hindrance effects.^51^ However, translating these subtle changes in outer-surface charge density into robust and quantitative readout signals for trace-level analyte detection remains a formidable challenge.

**Scheme 1:**
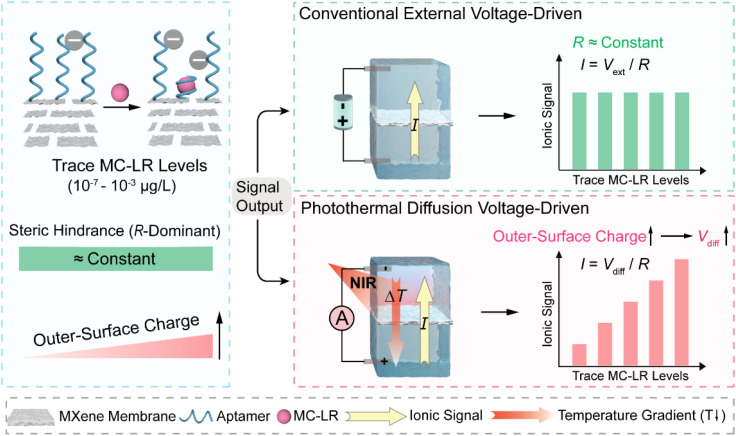
Schematic of ionic transport control in an MXene nanofluidic membrane at trace MC-LR levels in two driving modes. At 10^−7^–10^−3^ µg L^−1^, steric hindrance remains essentially constant, whereas outer-surface charge increases with MC-LR concentration. Because the steric hindrance-dominated resistance (*R*) baseline remains nearly invariant, the ionic current exhibits negligible changes, rendering external voltage-driven readout insensitive to trace levels of MC-LR. In contrast, in the photothermal diffusion voltage-driven mode, near-infrared (NIR) illumination generates a temperature gradient (Δ*T*) and a photothermal diffusion voltage (*V*_diff_) that is sensitively regulated by the outer-surface charge. Binding of MC-LR to outer-surface aptamers modulates the K^+^ transference number and thereby amplifies minute charge variations into substantial changes in *V*_diff_, enabling ultrasensitive detection of trace MC-LR.

Here, we develop a photothermal diffusion voltage-driven sensing strategy as an alternative to conventional external voltage-driven approaches to enable ultratrace-level detection in nanochannels. A stacked MXene nanofluidic membrane is employed as the device platform, in which outer-surface charge is transduced through a photothermal diffusion voltage (*V*_diff_) rather than relying solely on resistance changes, as illustrated in Scheme 1. By exploiting the near-infrared (NIR) photothermal response of MXene, a stable transmembrane temperature gradient (Δ*T*) is established across K^+^-permselective lamellar interlayer nanochannels within the membrane, creating a non-isothermal mode in which the ionic response is governed by outer-surface charge-dependent ion permselectivity. Direct comparison with the conventional external voltage-driven mode under a fixed external bias (*V*_ext_) on the same membrane device decouples the contributions of steric hindrance and outer-surface charge, revealing that the latter dominates the ionic response to ultratrace microcystin-LR (MC-LR). Combined experiments and numerical simulations further clarify how aptamer-mediated modulation of outer-surface charge reshapes ion transport characteristics and yields substantial signal gain. Consequently, this strategy enables quantitative detection of MC-LR down to 1 × 10^−7^ µg L^−1^ with sensitivity enhanced by more than 10^5^-fold compared with conventional external voltage-driven measurements, establishing outer-surface-charge-modulated photothermal diffusion voltage as a general route for actively regulating ionic transport in solid-state nanochannels.

## Results and discussion

### Fabrication and characterization of an outer-surface functionalized MXene nanofluidic membrane

**Fig. 1:**
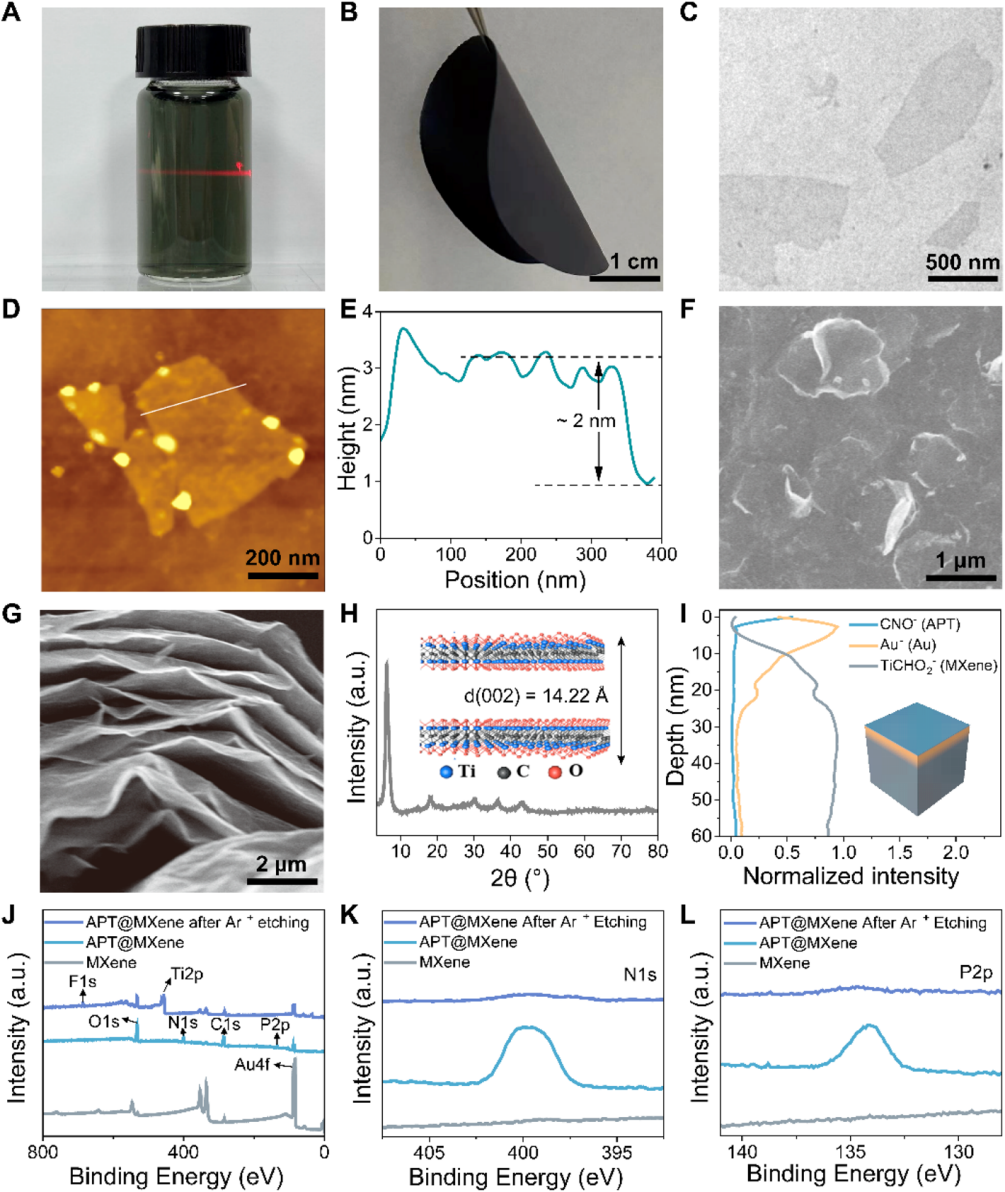
Characterization of the as-prepared MXene nanofluidic membrane and the outer-surface aptamer-functionalized membrane (APT@MXene). (A) Dispersion of MXene nanosheets exhibiting the characteristic Tyndall effect. (B) Photograph of the freestanding MXene membrane. (C) TEM, (D) AFM images of MXene nanosheets and (E) the corresponding height profile of the analyzed area. (F) Top-view and (G) cross-section view of SEM images of the MXene membrane. (H) XRD pattern of the MXene membrane. Interlayer spacing: *d*(002) = 14.22 Å. (I) Depth distribution of functional elements in APT@MXene, characterized by ToF-SIMS. The inset shows the corresponding 3D reconstruction based on the ToF-SIMS intensity distribution. (J) XPS survey spectra and high-resolution (K) N 1s and (L) P 2p XPS spectra of MXene, APT@MXene, and APT@MXene after Ar^+^ sputtering (target depth: ∼30 nm). These results confirmed the successful construction of the MXene nanofluidic membrane with aptamers exclusively immobilized on the outer surface.

The Ti_3_C_2_T_*x*_ MXene nanosheets were initially obtained by *in situ* etching of the MAX phase (Ti_3_AlC_2_) with an etchant composed of HCl and LiF, as previously reported.^52^ This process selectively removed the Al layer while simultaneously increasing the interlayer spacing (Fig. S1). As illustrated in Fig. 1A, the resulting colloidal suspension of exfoliated MXene nanosheets displayed a distinct Tyndall effect, signifying high dispersibility. Transmission electron microscopy (TEM) images revealed nearly transparent MXene nanosheets with pristine surfaces and large dimensions, which are suitable for constructing lamellar interlayer nanochannels in a nanofluidic membrane (Fig. 1C). Atomic force microscopy (AFM) analyses further determined the lateral dimensions of the MXene nanosheets that ranged from 200 to 500 nm, with an average thickness of approximately 2 nm (Fig. 1D and E). Subsequently, the nanosheets were restacked into a flexible, black-gray membrane through vacuum-assisted filtration (Fig. S2 and 1B). Despite its hydrophilic nature, the free-standing MXene membrane remained stable in water for up to one week under dark conditions, demonstrating mechanical robustness for biosensor applications (Fig. S3A). In addition, XRD characterization after immersion for 1, 2, and 7 days showed a well-defined (002) reflection without a noticeable shift toward lower 2*θ*, suggesting no evident irreversible interlayer expansion or structural degradation (Fig. S3B). Consistently, the ionic current responses under both external voltage-driven and photothermal diffusion voltage-driven operation remained stable over 7 days, supporting operational integrity under the sensing conditions (Fig. S3C and D). Scanning electron microscopy (SEM) observations confirmed that the MXene membrane consisted of stacked nanosheet layers, resulting in a characteristic lamellar microstructure (Fig. 1F and G). The slit-like gaps formed between nanosheets serve as subnanometer-scale fluidic nanochannels. Additionally, a distinct (002) diffraction peak at 6.22° was observed in the X-ray diffraction (XRD) pattern, corresponding to an interlayer spacing *d*(002) of 14.22 Å according to Bragg's law (Fig. 1H). Considering the theoretical thickness of a single MXene layer (∼9.8 Å), the effective interlayer spacing (ion diffusion space) within the MXene nanochannels was calculated to be approximately 4.42 Å, supporting efficient ion transport under confinement. To selectively modify the aptamer on the outer surface of the nanochannels (APT@MXene), a gold layer was deposited onto one side of the membrane using physical vapor deposition, as described in our previous work.^39^ The aptamer for MC-LR, functionalized with sulfhydryl groups, serves as a probe and is subsequently immobilized on the outer surface of the MXene membrane *via* Au–S bonding. Time of flight secondary ion mass spectrometry (ToF-SIMS) was employed to determine the distribution of the aptamer along APT@MXene. A threshold of 5% peak normalized intensity (indicated by the black dashed line) was applied to define the presence of elements. The Au region was measured at approximately 10 nm, consistent with the deposition thickness (Fig. 1I). In contrast, ToF-SIMS analysis revealed negligible aptamer penetration (effectively 0 nm) from the outermost membrane, confirming its predominant localization on the Au-deposited outer surface. X-ray photoelectron spectroscopy (XPS), coupled with argon ion sputtering, was employed to confirm the successful fabrication of APT@MXene. Spectra were acquired before and after a single Ar-ion sputtering step to a target depth of ∼30 nm. As depicted in Fig. 1J, the inherent O and C elements corresponded to the original MXene structure and its terminal functional groups. Following aptamer grafting, newly observed N 1s (399.8 eV) and P 2p (133.9 eV) peaks were ascribed to the incorporation of the aptamer. After argon ion sputtering, the N 1s and P 2p signals disappeared, while Ti 2p and F 1s signals appeared, further confirming that the aptamer was immobilized on the outer surface of the MXene and subsequently removed by sputtering. Detailed changes in the N 1s and P 2p signals under different conditions are also clearly illustrated in Fig. 1K and L. Taken together, the ToF-SIMS depth profile and the XPS sputtering results consistently indicate that the aptamer is predominantly localized on the Au-deposited outer surface. Additionally, LSCM analysis confirmed successful surface functionalization using the DCFH-labeled aptamer. Strong green fluorescence was observed only after immobilization (Fig. S4). The limited fluorescence penetration depth (∼2.8 µm), compared to the membrane thickness (∼20 µm), further verified that the aptamer was primarily localized on the outer surface. Furthermore, confocal fluorescence images collected from four randomly selected locations on the same membrane showed spatially homogeneous signals, and RuHex chronocoulometry yielded consistent aptamer surface coverage across the membrane (Fig. S5). Together, these results establish an outer-surface-functionalized nanochannel platform (APT@MXene) for subsequent comparisons between conventional external voltage-driven and photothermal diffusion voltage-driven modes.

### Constraints of the conventional external voltage-driven mode for outer-surface charge modulation

**Fig. 2:**
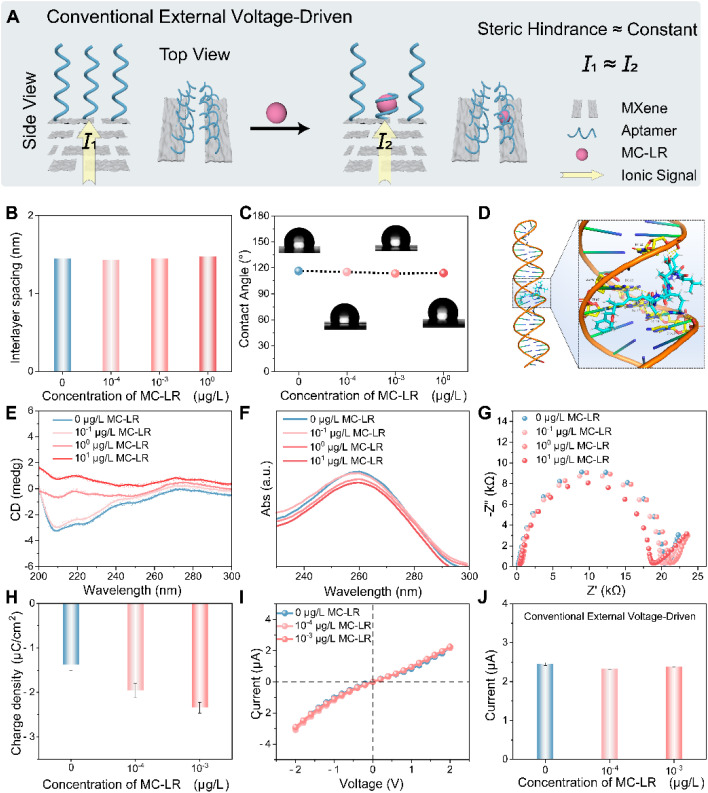
Ionic transport characteristics of APT@MXene in the conventional external voltage-driven mode. (A) Schematic illustration of steric hindrance arising from aptamer–MC-LR binding in the trace-concentration regime, where steric hindrance changes were minimal and the ionic current remained essentially unchanged. (B) Interlayer spacing measured by XRD and (C) surface wettability characterized by the static contact angle over a wide MC-LR concentration range. (D) Molecular docking prediction of the aptamer transition from an extended chain to a hairpin-like conformation. (E) CD spectra and (F) UV absorption spectra of the aptamer at different MC-LR concentrations. (G) Electrochemical impedance spectroscopy (EIS) spectra of APT@MXene at different MC-LR concentrations. (H) Chronocoulometry-derived outer-surface charge densities of APT@MXene at different MC-LR concentrations. (I) Representative *I*–*V* curves of APT@MXene under an external bias and (J) ionic currents recorded at −2 V for different MC-LR concentrations. Error bars represent standard deviations from three independent measurements. Overall, these results indicate that, at an applied voltage, ionic readout is constrained by an essentially unchanged steric hindrance-dominated resistance, such that small variations in outer-surface charge do not translate into a detectable change in current.

To establish a baseline and delineate the limits of conventional external voltage-driven transduction based on outer-surface functionalization, APT@MXene was first evaluated in the conventional external voltage-driven mode within the trace-concentration regime of MC-LR (Fig. 2A), where both steric hindrance and outer-surface-charge variations are expected to be subtle. Interlayer spacing (XRD, Fig. S6) and surface wettability (static contact angle) were quantified over a broad concentration range encompassing this regime and remained invariant within experimental uncertainty (Fig. 2B and C), indicating that nanochannel geometry and wetting do not appreciably affect ionic transport under these conditions. Molecular docking predicted that MC-LR binding induced a conformational transition of the aptamer from an extended chain to a hairpin-like structure, stabilized by groove-binding noncovalent interactions (Fig. 2D). Consistent with this prediction, CD spectra at high concentrations (≥1 µg L^−1^) showed decreased ellipticity at 209 nm and a red shift near 272 nm (Fig. 2E), and UV absorption exhibited hypochromicity at 260 nm (Fig. 2F), both hallmark signatures of DNA-aptamer folding. Importantly, these spectral signatures were absent within the trace-concentration window, suggesting that target-induced steric modulation was negligible in the regime of interest. Surface ionic accessibility was further probed by electrochemical impedance spectroscopy (EIS) using the [Fe(CN)_6_]^3−^/[Fe(CN)_6_]^4−^ couple.^53^ Under these conditions, the charge-transfer resistance (*R*_ct_) served as a proxy for the ease with which the anionic probe reached electron-active sites on the MXene surface, integrating steric accessibility and local electrostatics. A discernible decrease in *R*_ct_ emerged only at high concentrations, consistent with substantial aptamer conformational rearrangements that reduced steric hindrance, while no meaningful change was detected in the trace-concentration window (Fig. 2G). In contrast, chronocoulometry demonstrated that the outer-surface charge density became progressively more negative with increasing MC-LR concentration, with values of −1.38, −1.95, and −2.34 µC cm^−2^, and the effect was detectable even at 10^−4^ µg L^−1^ (Fig. 2H). Despite this measurable increase in outer-surface charge density, the *I*–*V* characteristics remained essentially indistinguishable across conditions (Fig. 2I), and at a fixed bias of −2 V the ionic current readout (*I* = *V*_ext_/*R*) showed no statistically significant difference (Fig. 2J). Taken together, these observations indicated that, in the trace-concentration regime, ionic readout in the conventional external voltage-driven mode was constrained by an effectively unchanged steric-hindrance-dominated resistance baseline, such that small variations in outer-surface charge were insufficient to produce a measurable change in current.

### Amplification of outer-surface charge modulation in the photothermal diffusion voltage-driven mode

**Fig. 3:**
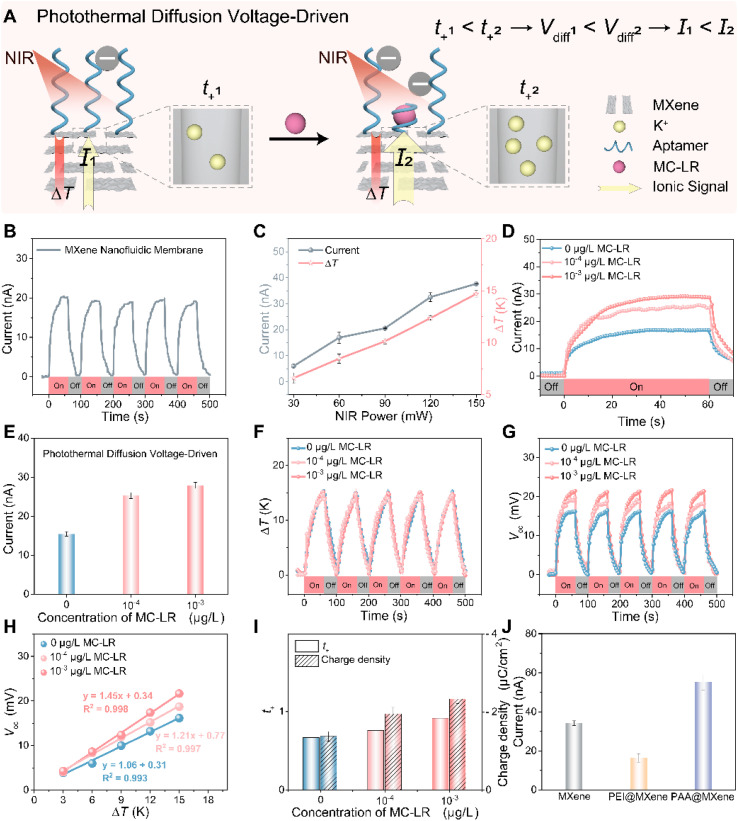
Ionic transport characteristics of APT@MXene in the photothermal diffusion voltage-driven mode. (A) Schematic illustration of ionic transport in APT@MXene nanochannels at different MC-LR concentrations, showing that increasing outer-surface charge raises the K^+^ transference number (*t*_+_), the photothermal diffusion voltage (*V*_diff_), and the resulting ionic current in the photothermal diffusion voltage-driven mode. (B) Representative photocurrent response of the MXene nanofluidic membrane under repeated 808 nm NIR (150 mW cm^−2^) on–off cycles. (C) Dependence of photocurrent and temperature gradient (Δ*T*) on incident NIR power. (D) Time-resolved photocurrent of APT@MXene at different MC-LR concentrations under 808 nm irradiation. (E) Photocurrent amplitudes of APT@MXene at varying MC-LR concentration. (F) Periodic Δ*T* upon repeated NIR irradiation and (G) the corresponding open-circuit photovoltage (*V*_oc_) response. (H) Linear correlation between *V*_oc_ and Δ*T*, used to extract the *t*_+_ at different MC-LR concentrations. (I) Extracted *t*_+_ values and outer-surface charge density of APT@MXene at different MC-LR concentrations. (J) Ionic current of the MXene nanofluidic membrane modified with PEI and PAA in photothermal diffusion voltage-driven mode. Error bars represent standard deviations of three independent experiments. Together, these results substantiate photothermal diffusion voltage-driven amplification of outer-surface charge modulation, in which trace level variations in outer-surface charge are translated into measurable changes in *V*_diff_ and consequently into readily detectable ionic current responses, whereas the conventional external voltage-driven readout remains nearly invariant over the same trace concentration range.

Building on the observation that ionic readout in the conventional external voltage-driven mode was insensitive to minor variations in outer-surface charge due to the invariant channel resistance, we implemented a photothermal diffusion voltage-driven strategy to investigate whether the ionic signal could be actively regulated in the trace-concentration regime. The MXene nanofluidic membrane bridged a two-compartment electrochemical cell filled with identical KCl solutions and Ag/AgCl electrodes, and no external bias was applied. Ionic current was generated and recorded by asymmetrically irradiating one side of the membrane with 808 nm NIR light, thereby creating a temperature gradient (Δ*T*) across the lamellar nanochannels and establishing the photothermal diffusion voltage-driven mode. Throughout this work, the irradiated side is defined as the hot side and the opposite side is defined as the cold side, with Δ*T* = *T*_hot_ − *T*_cold_. Upon NIR illumination, the membrane produced a rapid photocurrent strictly confined to the light-on periods. Repeated on/off cycling yielded stable and reproducible switching between high- and low-current states (Fig. 3B). Owing to the intrinsic photothermal response of MXene, both the ionic current and the nanochannel temperature gradient Δ*T* scaled approximately linearly with the incident irradiance (Fig. 3C). In contrast to the conventional external voltage-driven case, where the ionic current was invariant, the photothermal diffusion voltage-driven ionic current, measured at zero bias under fixed 808 nm illumination, increased with MC-LR concentration, rising from 15.4 nA (0 µg L^−1^) to 25.3 nA at 10^−4^ µg L^−1^ and 27.9 nA at 10^−3^ µg L^−1^ (Fig. 3D and E). These results indicated that photothermal diffusion voltage-driven mode effectively amplifies subtle outer-surface charge variations into measurable ionic current changes on the same APT@MXene device.

Mechanistically, previous studies have established that the asymmetric light stimulation of photoresponsive MXene-based nanofluidic system creates a temperature gradient across the membrane, which facilitates selective cation transport.^54,55^ This permselectivity is governed by surface charge, particularly at low electrolyte concentrations, due to the formation of electric double layers (EDLs) at the charged nanochannel interfaces. The resulting selective cation flux gives rise to a directional photothermal diffusion voltage (*V*_diff_) under the imposed temperature gradient Δ*T*. When the nanochannels are connected to symmetric Ag/AgCl electrodes, this diffusion voltage, together with the temperature-dependent redox voltages of the electrodes, manifests experimentally as an open-circuit photovoltage (*V*_oc_).^56,57^ Under non-isothermal conditions, the magnitudes of both *V*_diff_ and *V*_oc_ increase with enhanced cationic permselectivity. Therefore, we hypothesize that MC-LR binding modulates outer-surface charge, thereby altering cationic permselectivity and tuning *V*_diff_ to induce a detectable change in ionic current.

To substantiate this framework in our system, we measured the transmembrane ionic conductance of the MXene nanofluidic membrane across a wide range of KCl concentrations. As shown in Fig. S8, conductance increased linearly at higher concentrations (10^−2^ to 1 M) but deviated from bulk scaling and approached saturation at lower concentrations (10^−3^ to 10^−6^ M). This behavior reflected competition between bulk conduction and surface charge governed transport, influenced by the Debye length (*λ*_D_), which is inversely correlated with electrolyte concentration and determines ion selectivity according to Poisson–Boltzmann theory.^58–60^ Within this regime, we examined the photothermal diffusion voltage-driven ionic-current response of APT@MXene to 10^−3^ µg per L MC-LR across a range of KCl concentrations (10^−1^ M to 10^−5^ M). The largest ionic current change was observed at 10^−4^ M KCl (Fig. S10), whereas the conventional external voltage-driven system remained essentially unchanged across concentrations (Fig. S11). Having identified this optimal condition, we conducted a quantitative analysis to link the observed current modulation to changes in ionic selectivity. According to the non-isothermal ion-transport theory, the diffusion voltage *V*_diff_(*T*) and the experimentally measured open-circuit photovoltage *V*_oc_(*T*) can be expressed as^57^1
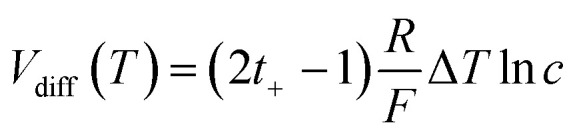
2
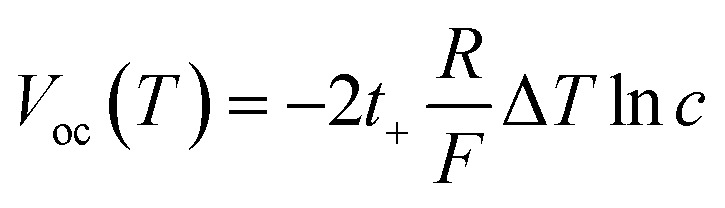
Here, *V*_diff_ is the photothermal diffusion voltage across the nanochannels under Δ*T*, and *V*_oc_ is the measured open-circuit voltage between the two Ag/AgCl electrodes. *t*_+_ represents the fraction of the total electric current carried by the cation. *R* represents the universal gas constant, and *F* is the Faraday constant. Δ*T* is the instantaneous temperature difference between the two reservoirs adjacent to the membrane, obtained simultaneously by infrared thermography from two fixed regions of interest in the hot- and cold-side reservoirs, respectively. *c* denotes the concentration of the KCl solution in the reservoirs. In the following analysis, we use the linear relationship between *V*_oc_ (*T*) and Δ*T* in eqn (2) to extract *t*_+_. As illustrated in Fig. 3F, under periodic 808 nm laser irradiation at 150 mW cm^−2^, APT@MXene exhibited a rapid, reversible thermal response. During each 60-second ‘On'’ cycle, the photo-induced temperature gradient (Δ*T*) quickly stabilized at approximately 14.8 K and returned to baseline during ‘Off’, consistently across all tested MC-LR concentrations. The modest gradient had a negligible effect on the aptamer, as the temperature on the non-illuminated side remained at 295.15 K. Concomitantly, an open-circuit photovoltage (*V*_oc_) was observed in all nanochannels under the same illumination (Fig. 3G), with five reproducible on–off cycles synchronized with Δ*T*. As the MC-LR concentration increased (0, 10^−4^, 10^−3^ µg L^−1^), the peak *V*_oc_ rose from 16.15 to 18.71 and 21.66 mV, respectively. To quantify the *V*_oc_–Δ*T* relationship, we matched *V*_oc_ and Δ*T* at the same time points during illumination and performed a linear regression using five representative pairs within Δ*T* = 3–15 K, which confirmed a linear scaling (Fig. 3H). According to eqn (2), with all other parameters held constant, the K^+^ transference number (*t*_+_) can be extracted from the slope of the fitted linear regression. The resulting *t*_+_ values were 0.67 for APT@MXene, 0.76 for APT@MXene + 10^−4^ µg per L MC-LR and 0.91 for APT@MXene + 10^−3^ µg per L MC-LR, respectively, indicating progressively enhanced K^+^ ion permselectivity. These trends closely track the increase in outer-surface charge density, implicating surface charge as a primary determinant of cation selectivity (Fig. 3I). Control experiments with ionic polyelectrolytes, including poly(ethyleneimine) (PEI, highly cationic) and poly(acrylic acid) (PAA, highly anionic), corroborated this conclusion. Upon outer-surface modification of the MXene nanofluidic membrane, PEI@MXene showed markedly reduced *V*_OC_ and ionic current compared with bare MXene, whereas PAA@MXene increased both responses (Fig. 3J), indicating that increasing negative surface charge strengthens cation permselectivity. Thus, we conclude that photothermal diffusion voltage-driven amplification of outer-surface charge modulation is realized in the MXene nanofluidic membrane. In this mode, K^+^ permselectivity acts as a transducer, converting subtle variations in outer-surface charge into substantial changes in the photothermal diffusion voltage and, consequently, the ionic current. In contrast, in conventional external voltage-driven mode, the channel resistance remains essentially constant, and the same trace-level charge variations produce no detectable current response.

**Fig. 4:**
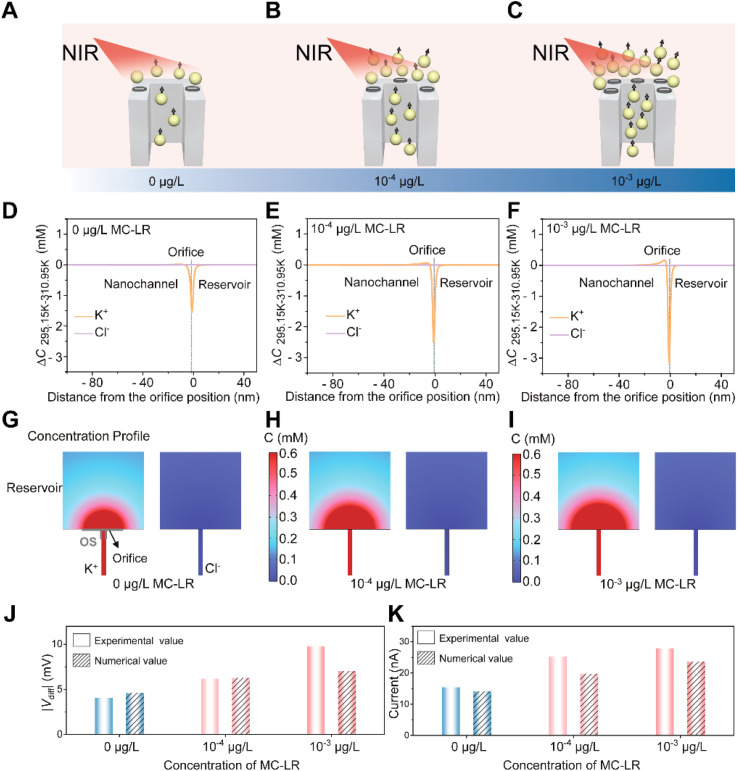
Numerical simulations of ion transport in the lamellar nanochannels in photothermal diffusion voltage-driven mode. (A–C) Predictive modeling of ion transport for three cases: APT@MXene, APT@MXene + 10^−4^ µg per L MC-LR and APT@MXene + 10^−3^ µg per L MC-LR, highlighting the effect of outer-surface charge. (D–F) Net concentration variation along the axial direction for the three cases under a temperature gradient of Δ*T* = 15 K. (G–I) Numerical simulations of ion concentration distributions for K^+^ (top) and Cl^−^ (bottom) at the nanochannel-reservoir interface for the three cases under Δ*T* = 15 K. (J) Magnitude of experimental and simulated photothermal diffusion voltages (|*V*_diff_|) for the three cases, obtained from experimental *t*_+_ and theoretical simulations, respectively. (K) Simulated and experimental ionic currents in the photothermal diffusion voltage-driven mode for the three cases. The simulations show that making the outer surface more negative strengthens cation-selective transport, deepens K^+^ depletion at the illuminated orifice, and increases both *V*_diff_ and the resulting ionic current, consistent with the experimental observations.

To gain deeper insight into the mechanism underlying ionic signal regulation, we performed a theoretical analysis of ion transport in the photothermal diffusion voltage-driven mode based on the Poisson–Nernst–Planck (PNP) framework and the Einstein–Stokes equation. The model explicitly distinguishes the negatively charged inner walls of the lamellar nanochannels (MXene nanosheets) from the outer surface region associated with aptamer immobilization and subsequent MC-LR treatment. The geometric design and corresponding boundary conditions are detailed in Fig. S13 and Table S5. From a thermodynamic perspective, directional ion transport in a light-responsive MXene nanofluidic membrane under asymmetric light stimulation can be rationalized by the dependence of Gibbs free energy on temperature, expressed as follows:^57^3
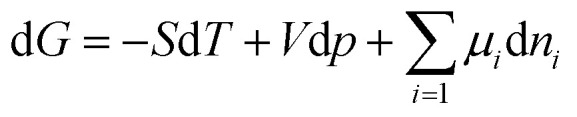
Here, *G*, *S*, *T*, *V*, and *p* represent the Gibbs free energy, entropy, temperature, volume, and pressure of the electrolyte solution, while *µ*_*i*_ and *n*_*i*_ denote the chemical potential and molar quantity of species *i*, respectively. Upon 808 nm irradiation, the illuminated reservoir is heated, establishing a transmembrane temperature gradient (Δ*T*) across the nanochannels. This non-isothermal condition modulates local electrochemical potentials and promotes directional ion redistribution between the two reservoirs. In the absence of illumination, electrostatic interactions in the negatively charged nanochannels enriched K^+^ and depleted Cl^−^, generating a concentration polarization layer at the reservoir-nanochannel interface. After applying Δ*T*, numerical simulations revealed pronounced K^+^ depletion at the nanochannel orifice, whereas the Cl^−^ profile remained nearly unchanged (Fig. 4D–F). The extent of K^+^ depletion increased with rising MC-LR concentrations and correlated with the experimentally derived outer-surface charge densities of −1.38, −1.95, and −2.34 µC cm^−2^, respectively. Because the inner-wall properties are identical across all simulations, these differences were attributed to variations in outer-surface charge density arising from aptamer–MC-LR interactions. A more negatively charged outer surface enhanced K^+^ enrichment within the nanochannels, thereby steepening the pre-existing concentration gradient. When this concentration gradient aligned with the Δ*T*, it reinforced directional K^+^ diffusion toward the side and amplifies the depletion at the orifice. Furthermore, Fig. 4G–I illustrate the simulated ion distributions for APT@MXene, APT@MXene + 10^−4^ µg per L MC-LR and APT@MXene + 10^−3^ µg per L MC-LR at a temperature difference of 15 K. In all cases, the nanochannels were predominantly occupied by K^+^, supporting directional cation transport. Importantly, the asymmetric distribution of K^+^ and Cl^−^ at the orifice induced localized electric-field variations, giving rise to the diffusion voltage *V*_diff_. As shown in Fig. 4J, the magnitude of the simulated *V*_diff_ exhibited the same increasing trend as the experimental values derived from *t*_+_, validating the diffusion voltage mechanism. Driven by this enhanced *V*_diff_, the simulated ionic currents aligned well with the experimental photothermal diffusion voltage-driven results (Fig. 4K). In contrast, simulations performed at a fixed external voltage (*V*_ext_ = −2 V) exhibited minimal variation (<10%) (Fig. S14), underscoring that the channel resistance remained dominated by invariant steric hindrance and confirming the insensitivity of the conventional external voltage-driven mode. Collectively, these analyses demonstrated that ion transport in the MXene nanofluidic membrane in the photothermal diffusion voltage-driven mode was governed by outer-surface charge controlled cation permselectivity, enabling minute charge changes to be amplified into measurable ionic signals *via* the tunable diffusion voltage.

### Ultratrace detection in the photothermal diffusion voltage-driven mode

**Fig. 5:**
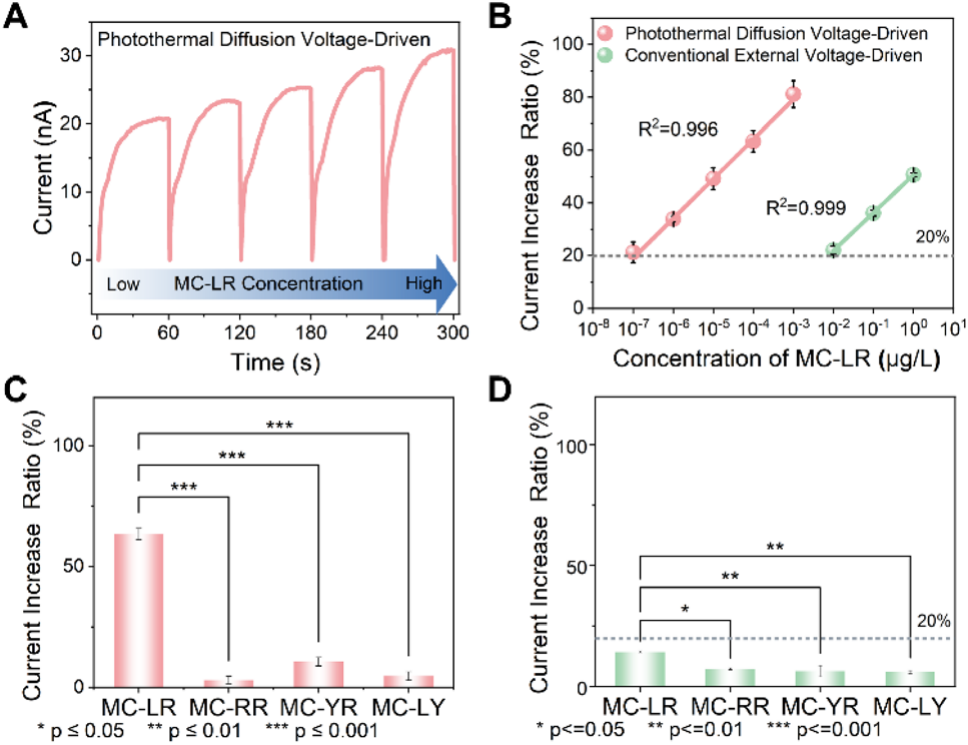
Sensitivity and specificity of the MXene nanofluidic aptasensor in two readout modes. (A) Time-dependent ionic current responses (*I*–*T* curves) of APT@MXene under periodic 808 nm NIR illumination (150 mW cm^−2^, 60 s) at increasing MC-LR concentrations. (B) Ultra-sensitive quantification of MC-LR in the two modes. Sensing specificity for MC-LR and its three analogous compounds in the (C) photothermal diffusion voltage-driven mode and (D) conventional external voltage-driven mode. Error bars represent standard deviations from three independent measurements.

Having established the mechanistic basis of photothermal diffusion voltage-driven amplification of outer-surface charge modulation, we investigated its potential for ultrasensitive detection of trace-level MC-LR. To quantify sensing performance, the target-induced ionic current increase ratio was defined as *f* = (*I*_APT@MXene+MC-LR_ − *I*_APT@MXene_)/*I*_APT@MXene_, where *I*_APT@MXene+MC-LR_ and *I*_APT@MXene_ represent the ionic current in the presence and absence of MC-LR with specific concentrations, respectively. A threshold of *f* > 20% was established as a reliable indicator of target recognition, whereas lower ratios were attributed to experimental noise or nonspecific adsorption. Upon exposure to increasing MC-LR concentrations, APT@MXene exhibited a progressive enhancement in ionic current in the photothermal diffusion voltage-driven mode (Fig. 5A). As illustrated in Fig. 5B, *f* increased markedly from 21.25% at 1 × 10^−7^ µg L^−1^ to 81.13% at 1 × 10^−3^ µg L^−1^, following a linear relationship with the logarithm of concentration: *f* = (0.15 × log[MC-LR] + 1.24) × 100% (*R*^2^ = 0.996). Based on the 20% threshold, the limit of detection (LOD) was determined to be 1 × 10^−7^ µg L^−1^, seven orders of magnitude below the World Health Organization (WHO) guideline value, thereby demonstrating the capacity for early-stage detection of trace MC-LR contamination. Compared with the conventional external voltage-driven mode, the photothermal diffusion voltage-driven strategy afforded a 10^5^-fold improvement in sensitivity. Additionally, Table S4 compares the performance of our photothermal diffusion voltage-driven strategy with that of other advanced techniques, highlighting its superior detection limit across a broad dynamic range. To evaluate the nanochannels' selectivity for MC-LR, we analyzed the current increase ratios induced by major microcystin analogs. The three predominant MC variants found in surface and drinking water sources, namely MC-RR, MC-YR, and MC-LY, were selected as potential interference factors. As shown in Fig. 5C, exposure to 1 × 10^−4^ µg L^−1^ of these four analogues yielded corresponding current increase ratios of 63.33%, 3.00%, 10.67%, and 4.67%, respectively. Statistical analysis confirmed significant discrimination (*p* < 0.001), underscoring the specificity of the photothermal diffusion voltage-driven mode, in stark contrast to the negligible selectivity observed in conventional external voltage-driven measurements (Fig. 5D). Finally, the applicability of the platform was validated in tap, lake, and mineral water samples using standard addition. Across spiking concentrations from 1 × 10^−6^ to 1 × 10^−3^ µg L^−1^, recoveries ranged from 96.76% to 109.30% with relative standard deviations (RSD) below 4.5% (Table 1), indicating negligible matrix interference and robust quantification accuracy. Collectively, these results demonstrate that photothermal diffusion voltage-driven amplification of outer-surface charge modulation enables precise and ultrasensitive control of ionic transport in the MXene nanofluidic membrane, establishing a reliable platform for early-stage environmental monitoring and food safety assessment of MC-LR.

**Table 1:** Detection of MC-LR in real water samples using a photothermal diffusion voltage-driven MXene nanofluidic aptasensor (*n* = 3)

Samples	Spiked (µg L^−1^)	Detected (µg L^−1^)	Recovery (%)	RSD (%)
Tap water	1 × 10^−3^	1.045 × 10^−3^	104.50	1.59
	1 × 10^−4^	1.042 × 10^−4^	104.20	0.84
	1 × 10^−5^	1.093 × 10^−5^	109.30	1.16
	1 × 10^−6^	1.042 × 10^−6^	104.20	4.50
Lake water	1 × 10^−3^	9.676 × 10^−4^	96.76	3.08
	1 × 10^−4^	1.091 × 10^−4^	109.10	3.52
	1 × 10^−5^	1.066 × 10^−5^	106.60	3.68
	1 × 10^−6^	9.939 × 10^−7^	99.39	2.99
Mineral water	1 × 10^−3^	1.064 × 10^−3^	106.40	1.36
	1 × 10^−4^	9.947 × 10^−5^	99.47	0.58
	1 × 10^−5^	1.061 × 10^−5^	106.10	2.27
	1 × 10^−6^	1.019 × 10^−6^	101.90	2.11

## Conclusions

In this work, we have established an outer-surface charge-modulated photothermal diffusion voltage-driven sensing strategy in an MXene nanofluidic membrane for ultratrace detection of MC-LR. Unlike conventional external voltage-driven readout that mainly converts target binding into ionic-current changes through resistance variation, our approach leverages a light-induced transmembrane temperature gradient to generate a diffusion voltage (*V*_diff_) whose magnitude is governed by K^+^ permselectivity and thus by outer-surface charge. Consequently, trace-level charge modulations, which produce little change in steric hindrance or the resistance baseline, are transduced into pronounced variations in *V*_diff_ and the zero-bias ionic current. This mechanism enables MC-LR quantification down to 1 × 10^−7^ µg L^−1^ with high selectivity against structural analogues and robust performance in real water samples. More broadly, these results demonstrate that outer-surface charge, rather than steric hindrance, can serve as a dominant and addressable control parameter for ionic transport in lamellar nanochannels, providing a general framework for light-regulated nanofluidic sensors and iontronic devices.

## Author contributions

Yihan Ma: conceptualization, formal analysis, investigation, resources, data curation and writing. Xinyi Yang: methodology, formal analysis, investigation and resources. Bingquan Qi: methodology and investigation. Xiaoping Yang: methodology and investigation. Zhengxu He: methodology and investigation. Ning Feng: methodology and investigation. Li Dai: methodology and investigation. Aiqing Zhang: conceptualization and supervision. Yu Huang: conceptualization, supervision, review & editing and project administration. Fan Xia: supervision and project administration.

## Conflicts of interest

There are no conflicts to declare.

## Supplementary Material

SC-017-D5SC10110G-s001

## Data Availability

The data supporting the findings can be found in the article and supplementary information (SI) and are available from the authors upon reasonable request. Supplementary information: experimental procedures, characterization data, and *I*–*V* curves. See DOI: https://doi.org/10.1039/d5sc10110g.

## References

[cit1] Huang J., Pan X., Yan N. (2024). Structural biology and molecular pharmacology of voltage-gated ion channels. Nat. Rev. Mol. Cell Biol..

[cit2] Li Y., Yuan T., Huang B., Zhou F., Peng C., Li X., Qiu Y., Yang B., Zhao Y., Huang Z., Jiang D. (2023). Structure of human NaV1.6 channel reveals Na+ selectivity and pore blockade by 4,9-anhydro-tetrodotoxin. Nat. Commun..

[cit3] He Z., Tu Y.-C., Tsai C.-W., Mount J., Zhang J., Tsai M.-F., Yuan P. (2025). Structure and function of the human mitochondrial MRS2 channel. Nat. Struct. Mol. Biol..

[cit4] Xin W., Fu J., Qian Y., Fu L., Kong X.-Y., Ben T., Jiang L., Wen L. (2022). Biomimetic KcsA channels with ultra-selective K+ transport for monovalent ion sieving. Nat. Commun..

[cit5] Leisle L., Lam K., Dehghani-Ghahnaviyeh S., Fortea E., Galpin J. D., Ahern C. A., Tajkhorshid E., Accardi A. (2022). Backbone amides are determinants of Cl− selectivity in CLC ion channels. Nat. Commun..

[cit6] Turney T. S., Li V., Brohawn S. G. (2022). Structural Basis for pH-gating of the K+ channel TWIK1 at the selectivity filter. Nat. Commun..

[cit7] Shang Z., Zhao J., Yang M., Xiao Y., Chu W., Xu S., Zhang X., Yi X., Lin M., Xia F. (2024). Precise control of transmembrane current via regulating bionic lipid membrane composition. Sci. Adv..

[cit8] Hoenig E., Han Y., Xu K., Li J., Wang M., Liu C. (2024). In situ generation of (sub) nanometer pores in MoS2 membranes for ion-selective transport. Nat. Commun..

[cit9] Zou K., Ling H., Wang Q., Zhu C., Zhang Z., Huang D., Li K., Wu Y., Xin W., Kong X.-Y., Jiang L., Wen L. (2024). Turing-type nanochannel membranes with extrinsic ion transport pathways for high-efficiency osmotic energy harvesting. Nat. Commun..

[cit10] Tsutsui M., Hsu W.-L., Hsu C., Garoli D., Weng S., Daiguji H., Kawai T. (2025). Transmembrane voltage-gated nanopores controlled by electrically tunable in-pore chemistry. Nat. Commun..

[cit11] Stuber A., Douaki A., Hengsteler J., Buckingham D., Momotenko D., Garoli D., Nakatsuka N. (2023). Aptamer Conformational Dynamics Modulate Neurotransmitter Sensing in Nanopores. ACS Nano.

[cit12] Zhang X., Dai Y., Sun J., Shen J., Lin M., Xia F. (2024). Solid-State Nanopore/Nanochannel Sensors with Enhanced Selectivity through Pore-in Modification. Anal. Chem..

[cit13] Li Z.-Q., Huang L.-Q., Wang K., Xia X.-H. (2024). Developing Solid-State Single-, Arrayed-, and Composite-Nanopore Sensors for Biochemical Sensing Applications. Acc. Mater. Res..

[cit14] Wang X., Liu Y., Ren R., Edel J. B., Ivanov A. P. (2025). Single-Molecule Protein Profiling Using Nanopores and Dimeric Aptamer-Modified DNA Carriers. Angew. Chem., Int. Ed..

[cit15] Arwani R. T., Tan S. C. L., Sundarapandi A., Goh W. P., Liu Y., Leong F. Y., Yang W., Zheng X. T., Yu Y., Jiang C., Ang Y. C., Kong L., Teo S. L., Chen P., Su X., Li H., Liu Z., Chen X., Yang L., Liu Y. (2024). Stretchable ionic–electronic bilayer hydrogel electronics enable in situ detection of solid-state epidermal biomarkers. Nat. Mater..

[cit16] Wang C., Li J., Li X., Li W., Li Y., Huang Y., Wang C., Liu Z., Wang M., Chen N., Chen M., Pan L., Zhang F., Bi J., Li L., Hu W., Chen X. (2025). Bio-inspired organic electrosense transistor for impalpable perception. Sci. Adv..

[cit17] Cheng W., Wang X., Xiong Z., Liu J., Liu Z., Jin Y., Yao H., Wong T.-S., Ho J. S., Tee B. C. K. (2023). Frictionless multiphasic interface for near-ideal aero-elastic pressure sensing. Nat. Mater..

[cit18] Huang Y., Liu L., Luo C., Liu W., Lou X., Jiang L., Xia F. (2023). Solid-state nanochannels for bio-marker analysis. Chem. Soc. Rev..

[cit19] Dauphin-Ducharme P., Arroyo-Currás N., Plaxco K. W. (2019). Correction to “High-Precision Electrochemical Measurements of the Guanine-, Mismatch-, and Length-Dependence of Electron Transfer from Electrode-Bound DNA Are Consistent with a Contact-Mediated Mechanism”. J. Am. Chem. Soc..

[cit20] Leung K. K., Gerson J., Emmons N., Heemstra J. M., Kippin T. E., Plaxco K. W. (2024). The Use of Xenonucleic Acids Significantly Reduces the In Vivo Drift of Electrochemical Aptamer-Based Sensors. Angew. Chem., Int. Ed..

[cit21] Zhao C., Hou J., Hill M., Freeman B., Wang H., Zhang H. (2023). Enhanced Gating Effects in Responsive Sub-nanofluidic Ion Channels. Acc. Mater. Res..

[cit22] Li X., Jiang G., Jian M., Zhao C., Hou J., Thornton A. W., Zhang X., Liu J. Z., Freeman B. D., Wang H., Jiang L., Zhang H. (2023). Construction of angstrom-scale ion channels with versatile pore configurations and sizes by metal-organic frameworks. Nat. Commun..

[cit23] Liu P., Kong X.-Y., Jiang L., Wen L. (2024). Ion transport in nanofluidics under external fields. Chem. Soc. Rev..

[cit24] Zhu C., Wu Y., Li X., Li X., Liang X., Jiang L., Wen L. (2025). Engineered Nanofluidics for Molecular Recognition and Physical Perception. Angew. Chem., Int. Ed..

[cit25] Zhang X., Cai H., Hu T., Lin M., Dai Y., Xia F. (2025). DNA-Functionalized Solid-State Nanochannels with Enhanced Sensing. Acc. Mater. Res..

[cit26] Nova I. C., Ritmejeris J., Brinkerhoff H., Koenig T. J. R., Gundlach J. H., Dekker C. (2024). Detection of phosphorylation post-translational modifications along single peptides with nanopores. Nat. Biotechnol..

[cit27] Wang R., Zhang Y., Ma Q. D. Y., Wu L. (2024). Recent advances of small molecule detection in nanopore sensing. Talanta.

[cit28] Li X., Zhai T., Gao P., Cheng H., Hou R., Lou X., Xia F. (2018). Role of outer surface probes for regulating ion gating of nanochannels. Nat. Commun..

[cit29] Zhu Z., Wang D., Tian Y., Jiang L. (2019). Ion/Molecule Transportation in Nanopores and Nanochannels: From Critical Principles to Diverse Functions. J. Am. Chem. Soc..

[cit30] Liang L., Qin F., Wang S., Wu J., Li R., Wang Z., Ren M., Liu D., Wang D., Astruc D. (2023). Overview of the materials design and sensing strategies of nanopore devices. Coord. Chem. Rev..

[cit31] Sun T., Zhao Z., Liu W., Xu Z., He H., Ning B., Jiang Y., Gao Z. (2020). Development of sandwich chemiluminescent immunoassay based on an anti-staphylococcal enterotoxin B Nanobody–Alkaline phosphatase fusion protein for detection of staphylococcal enterotoxin B. Anal. Chim. Acta.

[cit32] Zhang W.-Q., Tu Y.-D., Liu H., Liu R., Zhang X.-J., Jiang L., Huang Y., Xia F. (2024). A Single Set of Well-Designed Aptamer Probes for Reliable On-site Qualitative and Ultra-Sensitive Quantitative Detection. Angew. Chem., Int. Ed..

[cit33] Lin M., Yang M., Xiao Y., Zhao J., Shang Z., Liu X., Wang L., Pan J., Yi X., Zhang X., Xia F. (2025). Graphene Oxide Nanofluidic Ion Channels with Two-Gene Rolling Circle Amplification for Ultrasensitive and Specific Detection of SARS-CoV-2. Anal. Chem..

[cit34] Loha K., Boonkoom T., Pitakjakpipop H., Alam I., Treetong A., Boonbanjong P., Chatnuntawech I., Teerapittayanon S., Keyser U. F., Schulte A., Japrung D. (2025). Structural and Kinetic Profiling of Rolling Circle Amplification via Solid-State Nanopore Sensing Using miR-21 as a Model. ACS Sens..

[cit35] Shrikrishna N. S., Gandhi S. (2025). Nanopore-based sensing for biomarker detection: from fundamental principles to translational diagnostics. J. Nanobiotechnol..

[cit36] Li P., Zhao J., Liang D., Peng C., Zhu J., Yeom B., Wang Z., Zhao Y., Ma W. (2025). Construction of Biomimetic Nanochannel, Property Regulation, and Biomarker Detection. Small.

[cit37] Nestorovich E. M., Rostovtseva T. K., Bezrukov S. M. (2003). Residue Ionization and Ion Transport through OmpF Channels. Biophys. J..

[cit38] Bajaj H., Acosta Gutierrez S., Bodrenko I., Malloci G., Scorciapino M. A., Winterhalter M., Ceccarelli M. (2017). Bacterial Outer Membrane Porins as Electrostatic Nanosieves: Exploring Transport Rules of Small Polar Molecules. ACS Nano.

[cit39] Ma Q., Li Y., Wang R., Xu H., Du Q., Gao P., Xia F. (2021). Towards explicit regulating-ion-transport: nanochannels with only function-elements at outer-surface. Nat. Commun..

[cit40] Si Z., Xu H., Lin M., Jiang Y., Du Q., Ma H., Liang H., Gao P., Xia F. (2022). Polydopamine-Induced Modification on the Highly Charged Surface of Asymmetric Nanofluidics: A Strategy for Adjustable Ion Current Rectification Properties. Anal. Chem..

[cit41] Zhang W., Chen M., Ma Q., Si Z., Jin S., Du Q., Zhang L., Huang Y., Xia F. (2024). Role of Outer Surface Probes on Bullet-Shaped Asymmetric Solid-State Nanochannels for Lysozyme Protein Sensing. Anal. Chem..

[cit42] Dai L., Zhang W.-Q., Ding D., Luo C., Jiang L., Huang Y., Xia F. (2024). Outer-Surface Functionalized Solid-State Nanochannels for Enhanced Sensing Properties: Progress and Perspective. ACS Nano.

[cit43] Ma L., Liu Z., Ai B., Man J., Li J., Wu K., Qiu Y. (2024). Ion transport through short nanopores modulated by charged exterior surfaces. J. Chem. Phys..

[cit44] Qiao Y., Hu J.-J., Hu Y., Duan C., Jiang W., Ma Q., Hong Y., Huang W. H., Xia F., Lou X. (2023). Detection of Unfolded Cellular Proteins Using Nanochannel Arrays with Probe-Functionalized Outer Surfaces. Angew. Chem., Int. Ed..

[cit45] Hu J.-J., Jiang W., Qiao Y., Ma Q., Du Q., Jiang J.-H., Lou X., Xia F. (2023). Enzyme Regulating the Wettability of the Outer Surface of Nanochannels. ACS Nano.

[cit46] Zhang C., Zhang X., Zhang H., Gong Z., Ren X., Guo M., Qiu Y. (2025). Modulation of electroosmotic flow through short nanopores by charged exterior surfaces. Electrochim. Acta.

[cit47] Liu L., Ding D., Luo C., Wang X., Tian Y., Liu S.-C., Lou X., Huang Y., Mao L., Xia F. (2025). Nanochannels with Varied Outer Surface Charges for Protein Discrimination. Anal. Chem..

[cit48] Chen Y., Li X., Yue X., Yu W., Shi Y., He Z., Wang Y., Huang Y., Xia F., Li F. (2025). Sub-femtomolar drug monitoring via co-calibration mechanism with nanoconfined DNA probes. Nat. Commun..

[cit49] Ai B., Gong Z., Zhang H., Lu Z., Sui T., Qiu Y. (2026). Modulation of Ion Distributions and Electroneutrality inside Nanopores by Ion Transport under Electric Fields. ACS Appl. Mater. Interfaces.

[cit50] Zhang H., Ai B., Gong Z., Sui T., Siwy Z. S., Qiu Y. (2025). Ion Transport through Differently Charged Nanoporous Membranes: From a Single Nanopore to Multinanopores. Anal. Chem..

[cit51] Lin M., Zhao J., Yi X., Xiao Y., Shang Z., Xu L., Lei X., Pan J., Huang Y., Zhang X., Xia F. (2025). Role of the real first interface in regulating ionic signal of nanochannels. Nat. Commun..

[cit52] Ding L., Xiao D., Lu Z., Deng J., Wei Y., Caro J., Wang H. (2020). Oppositely Charged Ti3C2T MXene Membranes with 2D Nanofluidic Channels for Osmotic Energy Harvesting. Angew. Chem., Int. Ed..

[cit53] Nakatsuka N., Faillétaz A., Eggemann D., Forró C., Vörös J., Momotenko D. (2021). Aptamer Conformational Change Enables Serotonin Biosensing with Nanopipettes. Anal. Chem..

[cit54] Lao J., Wu S., Gao J., Dong A., Li G., Luo J. (2020). Electricity generation based on a photothermally driven Ti3C2Tx MXene nanofluidic water pump. Nano Energy.

[cit55] Yeom J., Choe A., Lee J., Kim J., Kim J., Oh S. H., Park C., Na S., Shin Y.-E., Lee Y., Ro Y. G., Kwak S. K., Ko H. (2023). Photosensitive ion channels in layered MXene membranes modified with plasmonic gold nanostars and cellulose nanofibers. Nat. Commun..

[cit56] Hong S., Zou G., Kim H., Huang D., Wang P., Alshareef H. N. (2020). Photothermoelectric Response of Ti3C2Tx MXene Confined Ion Channels. ACS Nano.

[cit57] Chen K., Yao L., Su B. (2019). Bionic Thermoelectric Response with Nanochannels. J. Am. Chem. Soc..

[cit58] Ma Q., Chu W., Nong X., Zhao J., Liu H., Du Q., Sun J., Shen J., Lu S.-M., Lin M., Huang Y., Xia F. (2024). Local Electric Potential-Driven Nanofluidic Ion Transport for Ultrasensitive Biochemical Sensing. ACS Nano.

[cit59] Ma L., An X., Song F., Qiu Y. (2022). Effective Charged Exterior Surfaces for Enhanced Ionic Diffusion through Nanopores under Salt Gradients. J. Phys. Chem. Lett..

[cit60] Ma L., Liu Z., Man J., Li J., Siwy Z. S., Qiu Y. (2023). Modulation mechanism of ionic transport through short nanopores by charged exterior surfaces. Nanoscale.

