# Supplementary material for: Outer-surface charge modulation of photothermal diffusion voltage enables ultrasensitive sensing in nanofluidic membranes
Source: Chem Sci. 2026 Feb 25;17(16):8148–59. doi: 10.1039/d5sc10110g (PMC12958142; doi:10.1039/d5sc10110g)
Supplement: SC-017-D5SC10110G-s001 [file SC-017-D5SC10110G-s001.pdf]

Supplementary Information (SI) for Chemical Science.
This journal is © The Royal Society of Chemistry 2026

Supporting Information of

Outer-surface charge modulation of photothermal diffusion voltage enables ultrasensitive sensing in nanofluidic membranes

*Yihan Ma,[†a] Xinyi Yang,[†a] Bingquan Qi,[b] Xiaoping Yang,[a] Zhengxu He,[b] Ning Feng,[a] Li Dai,[b] Aiqing Zhang,[a] Yu Huang,[*b] and Fan Xia[b]*

a Key Laboratory of Catalysis and Energy Materials Chemistry of Ministry of Education & Hubei Key Laboratory of Catalysis and Materials Science, South-Central Minzu University, Wuhan 430074, China

b State Key Laboratory of Geomicrobiology and Environmental Changes, Engineering Research Center of Nano-Geomaterials of Ministry of Education, Faculty of Materials Science and Chemistry, China University of Geosciences, Wuhan 430074, China

† These authors contributed equally to this work

Corresponding Authors. E-mail: yuhuang@cug.edu.cn

Materials and Methods

Chemicals and reagents

All chemicals were used as received without further purification. Microcystins (MC-LR, MC-RR, MC-YR, and MC-LY) were purchased from Eurofins Abraxis Inc. Lithium fluoride was sourced from Aladdin. Hydrochloric acid and potassium chloride were procured from the China National Pharmaceutical Group Corporation (Sinopharm). Ti_3AlC_2 was acquired from Shandong Xinyan New Material Technology Co., Ltd. Mixed fiber membranes were supplied by Shanghai Xingya Purification Material Factory. Tris (tris(hydroxymethyl)aminomethane), poly(acrylic acid) (PAA, MW ~ 5000), and poly(ethylene imine) (PEI) were purchased from Guangzhou Saiguo Biotechnology Co., Ltd. The single-stranded DNA aptamer (5′-HS–C6-GGCGCCAAACAGGACCACCATGACAATTACCCATACCACCTCATTATGCC CCATCTCCGC-3′) was synthesized by Sangon Biotech (Shanghai) Co., Ltd. All solutions were prepared using deionized water with a resistivity of 18.2 MΩ·cm.

Fabrication of MXene nanofluidic membrane

To synthesize the MXene nanosheets, a mixture of 1.4 g lithium fluoride and 16 mL hydrochloric acid (36%) was stirred at 35 °C for 5 minutes, after which 0.7 g Ti_3AlC_2 was added. The temperature was raised to 45 °C, and the reaction mixture was stirred for 2 days. The resulting product was washed three times with 1 M hydrochloric acid and then water until the pH of the supernatant reached neutrality. The product was then ultrasonicated for 30 minutes under a nitrogen atmosphere and centrifuged at 3,500 r/min for 30 minutes. The supernatant obtained was the MXene nanosheets aqueous

solution. To prepare MXene membranes, 40 mL of the MXene nanosheet aqueous solution (1 mg/mL) was sonicated for 10 minutes to ensure homogeneous dispersion. The dispersion was then subjected to vacuum filtration onto a mixed cellulose ester membrane (0.22 μm pore size, 50 mm diameter) and subsequently dried under vacuum at 60 °C for 8 hours. Next, a 2 nm chromium (Cr) layer and a 10 nm gold (Au) layer were sequentially deposited onto the membrane using the physical vapor deposition (PVD) method with an AE Nexdap PVD platform. APT@MXene was fabricated by introducing a 1 μM aptamer solution in Tris-HCl buffer (pH 7.4, 10 mM Tris, 500 mM NaCl, 1 mM $MgCl_2$) onto the above MXene membrane, incubating for 12 hours, and then washing with Tris-HCl buffer before air-drying at room temperature. For the accurate quantification of MC-LR, MC-LR solutions at various concentrations were prepared using deionized water and applied to the surface of APT@MXene for 2 hours. After washing with deionized water and air drying at room temperature, the resulting membrane device was prepared for subsequent testing and characterization.

Characterizations

SEM images were acquired using a field-emission scanning electron microscope (SU8010, Hitachi). Morphology of MXene nanosheets was characterized by a transmission electron microscope (TEM, TECNAL G2 20 S-TWIN) and an atomic force microscope (AFM, SPM-9700HT). The zeta potential of MXene nanosheets, the aptamer and the MC-LR in water were determined on a Zetasizer Nano ZSP instrument (DLS). Interlayer spacing was characterized by X-ray diffraction (XRD) using a D8 ADVANCE equipped with Cu Kα radiation (λ = 1.5406 Å). Elemental analysis was

conducted using an X-ray photoelectron spectrometer (XPS, EscalabXi^{+}) with an Al Kα X-ray source (1486.6 eV). For sputtering-assisted measurements, Ar-ion sputtering was performed at 2.0 keV with a Ta_2O_5-calibrated sputter rate of 0.10 nm s^{-1} for 300 s (target depth: ~30 nm). Time-of-flight secondary ion mass spectrometry (ToF-SIMS; IONTOF GmbH, Germany) was employed to analyze the spatial distribution of aptamer along the nanochannels. Confocal images before and after aptamer immobilization on the outer surface of the membrane were obtained using a confocal laser scanning microscope (Leica TCS SP8). Contact angle measurements were performed using an analyzer (DSA 100) by averaging data from three parallel measurements of a 10 μL water droplet on membrane surfaces.

Electrochemical impedance spectroscopy (EIS) was performed using a three-electrode configuration, with APT@MXene or APT@MXene + MC-LR as the working electrodes, an Ag/AgCl electrode as the reference electrode, and a platinum wire as the counter electrode. The EIS measurements were conducted in a solution containing 5 mM $[Fe(CN)_6]^{3-}$ and 0.1 M KCl at pH 7.0. An AC voltage amplitude of 5 mV was applied, and the frequency range was set from 0.01 Hz to 100 kHz.

The outer-surface charge density of the MXene nanofluidic membrane was determined by chronocoulometry. A three-electrode configuration was employed, consisting of the MXene membrane as the working electrode, an Ag/AgCl reference electrode, and a platinum wire counter electrode. The MXene membrane was first immersed in Tris-HCl buffer (10 mM Tris, pH 7.0), and chronocoulometric measurements were performed over a voltage range from +0.2 V to −0.6 V for 500 ms.

The experiment was then repeated after adding 50 μM RuHex to the Tris-HCl buffer. The integrated charge (Q) as a function of time (t) in chronocoulometry follows the integrated Cottrell equation.

$$Q = \frac{2nFAD_0^{1/2}C_0^*}{\pi^{1/2}}t^{1/2} + Q_{dl} + nFA\Gamma_0 \quad (1)$$

where n is the number of electrons transferred per molecule during reduction, F is the Faraday constant (C/mol), A is the electrode area (cm^2), D_0 is the diffusion coefficient (cm^2/s), and C_0^* is the bulk concentration (mol/cm^3). Q_{dl} represents the double-layer (capacitive) charge (C), and $nFA\Gamma_0$ corresponds to the charge arising from the reduction of adsorbed redox species with a surface coverage Γ_0 (mol/cm^2). The term Γ_0 denotes the surface excess and reflects the amount of redox-active species confined near the electrode surface.

Chronocoulometric data are typically presented as an Anson plot (Q vs. $t^{1/2}$). The intercept at $t = 0$ equals ($Q_{\mathrm{dl}} + nFA\Gamma_0$). Because Q_{dl} remains constant, the difference in intercepts obtained from measurements in the absence and presence of RuHex directly reflects the contribution of adsorbed redox species, allowing the surface charge density of the MXene nanochannel to be quantitatively evaluated.

The saturated surface excess of RuHeX can be converted to the grafting density of aptamer on the MXene membrane with the relationship, as equation (2).

$$\Gamma_{APT} = \Gamma_0 \frac{z}{m} N_A \quad (2)$$

where Γ_{APT} is the grafting density of aptamer on the MXene membrane in molecules/cm^2, m is the number of bases in the aptamer, z is the charge of the RuHeX (z = 3), and N_A is Avogadro's number.

Ionic current measurement

The MXene nanofluidic membrane was integrated into the center of a custom-made two-compartment electrochemical cell, which contained a pair of Ag/AgCl electrodes. For the conventional external voltage-driven system, current-voltage (*I-V*) characteristics across the nanochannels were recorded using a picoammeter (Keithley 6487). The external field was scanned from -2 V to 2 V at various KCl concentrations ranging from 10^{-4} to 0.1 M. For the photothermal diffusion voltage-driven system, one side of the membrane device was irradiated with a laser (808 nm, 150 mW/cm^2), and the resulting temperature changes were captured by an infrared camera (FLIR ONE PRO). Under localized light illumination, amperometric current-time (*I-t*) characteristics across the nanochannels at various KCl concentrations ranging from 10^{-4} to 0.1 M were recorded using the picoammeter, and open-circuit voltages (V_{oc}) were measured using an electrochemical workstation (CHI660E). All tests were conducted at 295.15 K, with at least 15 samples measured to obtain average values.

Detection of MC-LR in water samples

To assess the applicability of the photothermal diffusion voltage-driven MXene nanofluidic aptasensor in real-world samples, matrix effect analysis and water sample analysis were conducted using the standard addition method. Tap water, mineral water, and lake water samples from East Lake in Wuhan were filtered through 220 nm filters

to remove solid impurities and plankton, and then spiked with MC-LR concentrations ranging from 1×10^{-6} to 1×10^{-3} μg/L. These spiked solutions were used to replace the buffer solution containing MC-LR during the target recognition procedure between the probes and microcystins. To ensure reliability, all real water samples were tested in triplicate using the photothermal diffusion voltage-driven Mxene nanofluidic aptasensor. The electrochemical testing conditions were consistent with those described above.

Numerical Simulation

Ion transport in a representative MXene nanochannel (within the MXene nanofluidic membrane) was simulated using commercial COMSOL (version 6.2) multiphysics software. A two-dimensional nanochannel model was constructed as shown in **figure S12**, with a channel radius of r_{ch} = 1.4 nm and a length of L = 100 nm, which was connected to two reservoirs filled with aqueous KCl solution. The boundary conditions in numerical simulation are summarized in **Table S5**. The coupled Poisson-Nernst-Planck (PNP) and Einstein-Stokes equations are used to quantitatively describe the ionic mass transport process:

$$\nabla^2\phi = -\frac{\rho}{\varepsilon} = -\frac{F}{\varepsilon}\Sigma z_i c_i \quad (3)$$

$$\vec{J}_i = -D_i\left(\nabla c_i + \frac{z_i c_i F}{RT}\nabla\phi\right) \quad (4)$$

$$\nabla \cdot \vec{J}_i = 0 \quad (5)$$

$$D_i = \frac{k_B T}{6\pi\eta r} \quad (6)$$

where

φ [V]: electric potential.

ρ [C/m^3]: space charge density.

$\vec{J}_i$ [mol/(m^{2}s)]: ionic flux.

z_i [-]: charge number.

c_i [M]: ion concentration.

D_i [m^2/s]: diffusion coefficient.

ε = 78.5 [F/m]: dielectric constant of water.

F [C/mol]: Faraday constant.

R [J/K/mol]: gas constant.

k_B: Boltzmann constant.

T [K]: absolute temperature.

η [Pa*s]: the dynamic fluid viscosity of water, expressed by a built-in piecewise function in COMSOL.

r is the ion size (for K^+ /Cl^-, r = 0.3 nm);

i = + (for K^+).

i = - (for Cl^-).

Using suitable boundary conditions, the ion concentration distribution and voltage distribution in the orifice under this condition can be obtained by solving equations. (3-6). Finally, the transmembrane current of the nanochannel can be calculated by using equation (7).

$$I = F\int_S \left(\sum_{i=1}^{2} z_i \vec{J}_i\right) \cdot \vec{n} dS \qquad (7)$$

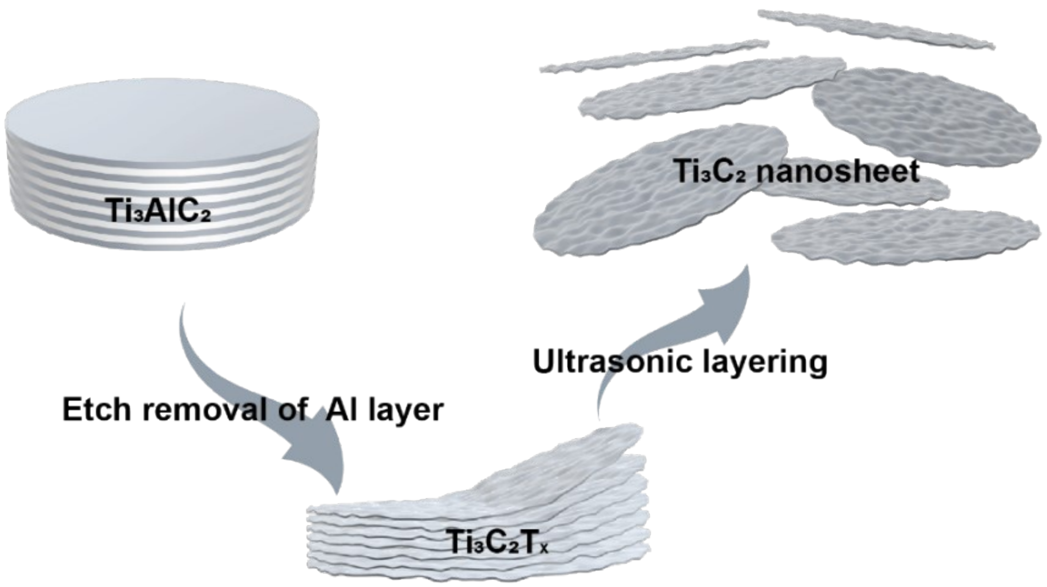

Figure S1 Schematic illustration of MXene nanosheets prepared by selectively etching the Ti_3AlC_2 phase.

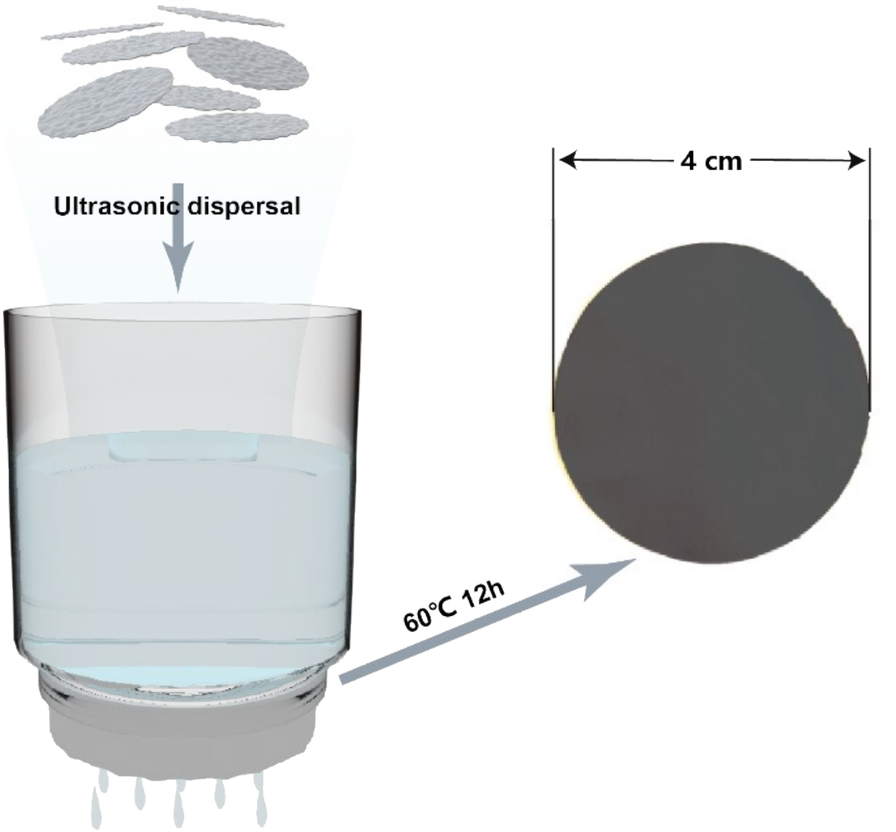

Figure S2 Schematic illustration of the preparation process of the MXene membrane.

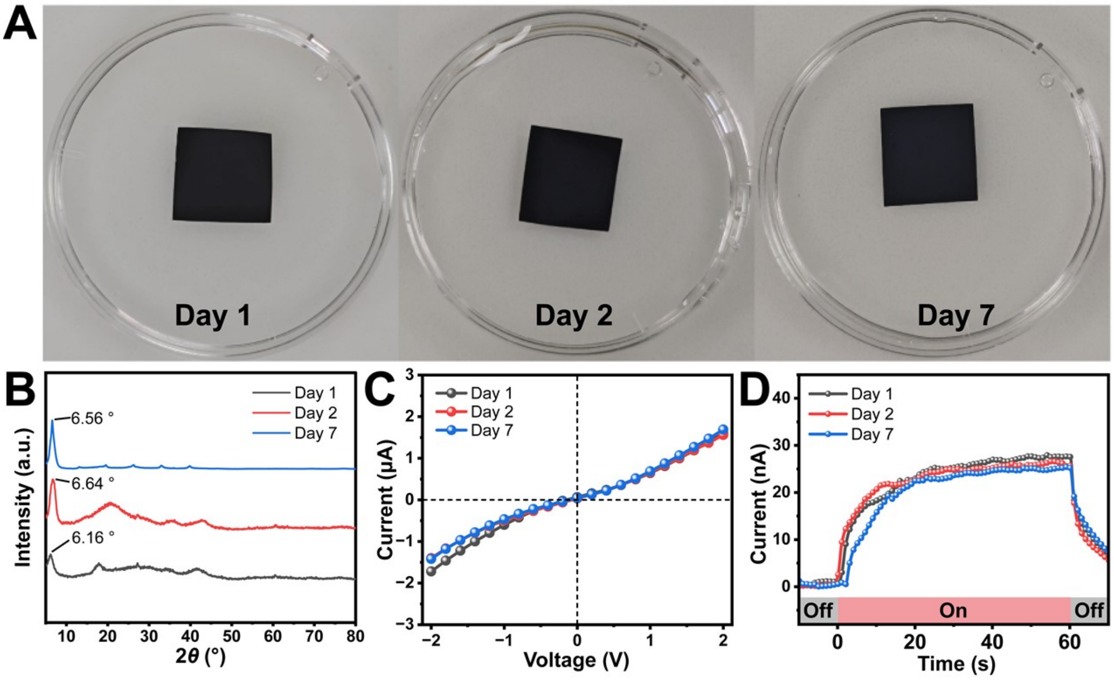

Figure S3 (A) Stability of the MXene membrane over a 7-day period (water immersion, dark storage, room temperature). (B) XRD patterns of the MXene membrane after immersion in water for 1, 2, and 7 days (measured after drying). (C) *I*-*V* characteristics under external voltage-driven operation and (D) time-resolved photocurrent under photothermal diffusion voltage-driven operation at Day 1, Day 2, and Day 7.

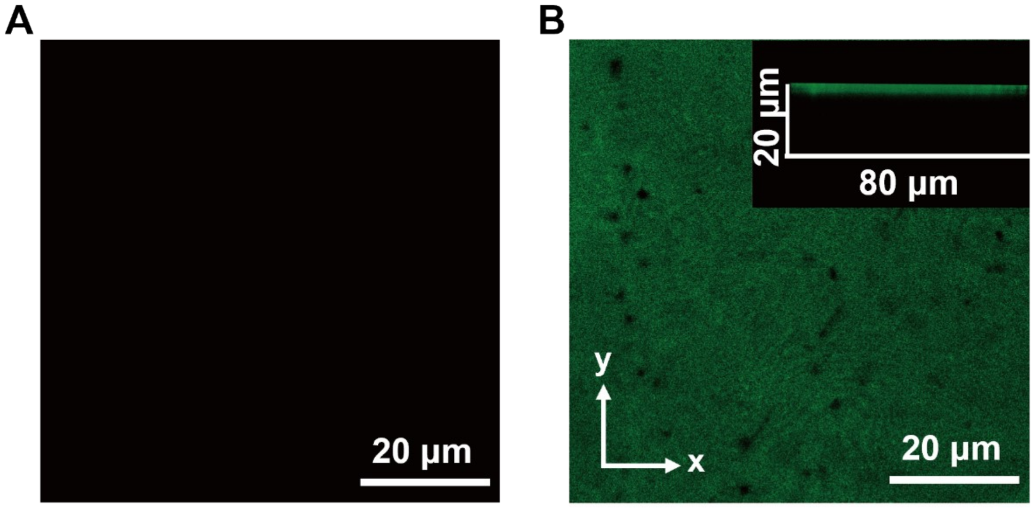

Figure S4 (A) Top-view LSCM image of MXene membrane. (B) Top-view and cross-sectional view of LSCM images of APT@MXene. The aptamer was labeled with the fluorescent dye FITC.

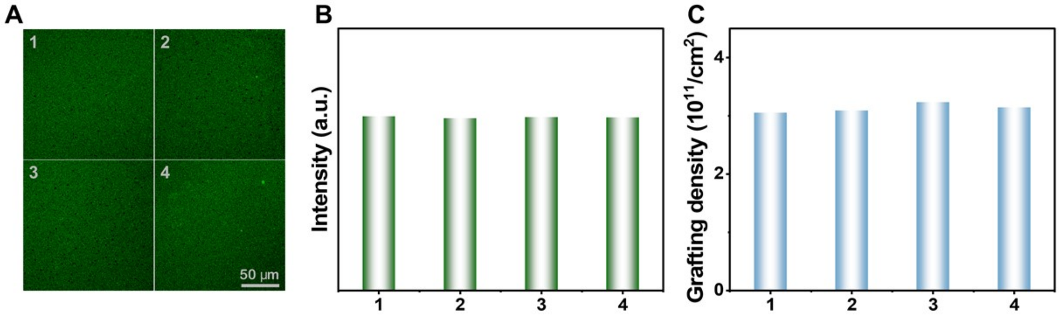

Figure S5. (A) Confocal fluorescence images of DCFH-labeled aptamer on the MXene membrane at four randomly selected locations on the same membrane. (B) Mean fluorescence intensity quantified from 1-4 (RSD = 0.4%, n = 4). (C) Aptamer grafting density quantified by RuHex-CC at four locations on the same membrane (RSD = 2.5%, n = 4).

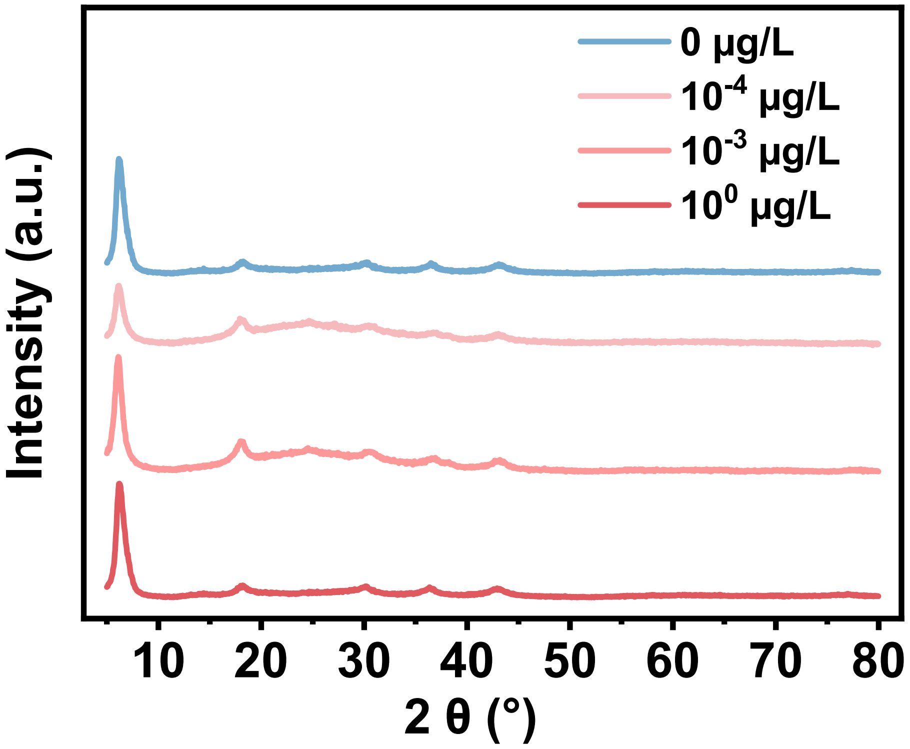

Figure S6 XRD patterns of APT@MXene combined with 0 μg/L , 10^{-4} μg/L , 10^{-3} μg/L, 10^{0} μg/L MC-LR.

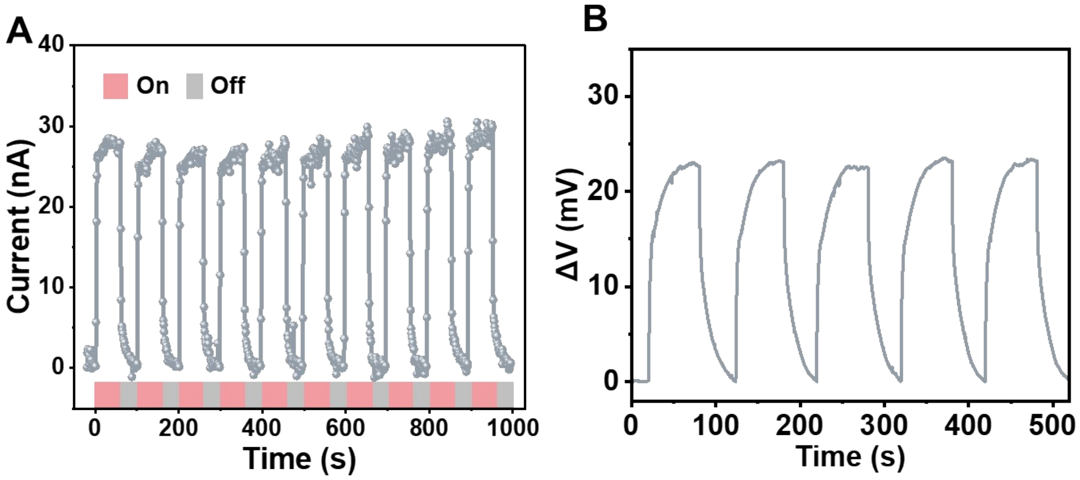

Figure S7. Representative (A) photocurrent response and (B) open-circuit photovoltage (V_{oc}) of the MXene nanofluidic membrane under repeated 808 nm NIR (150 mW/cm²) on-off cycles.

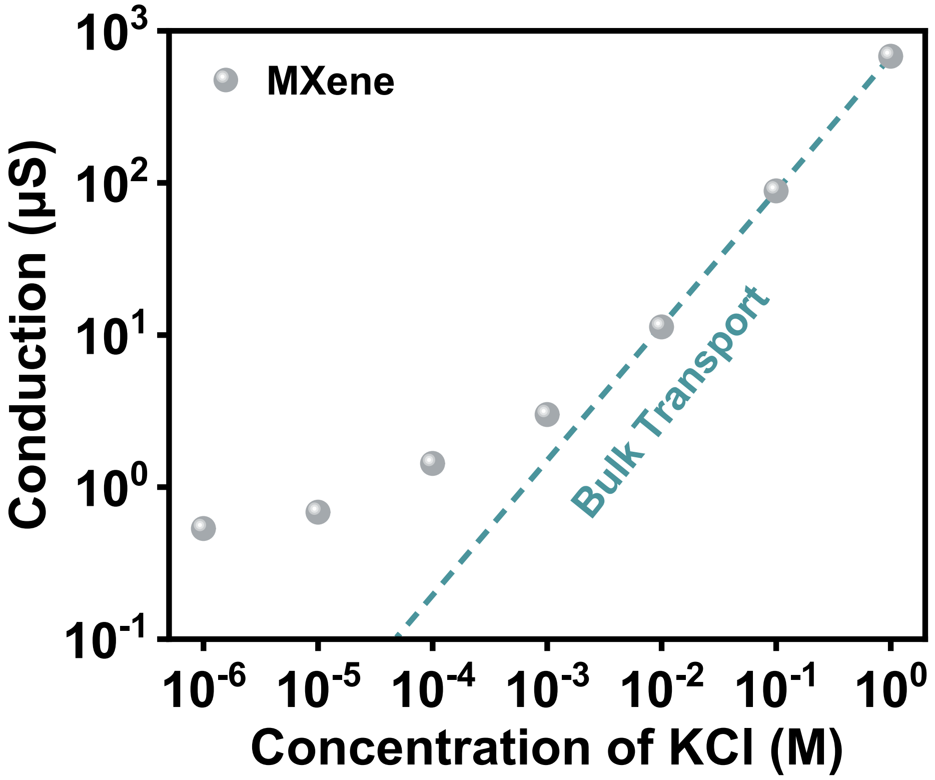

Figure S8 Ionic conductance of MXene membrane under different KCl concentrations.

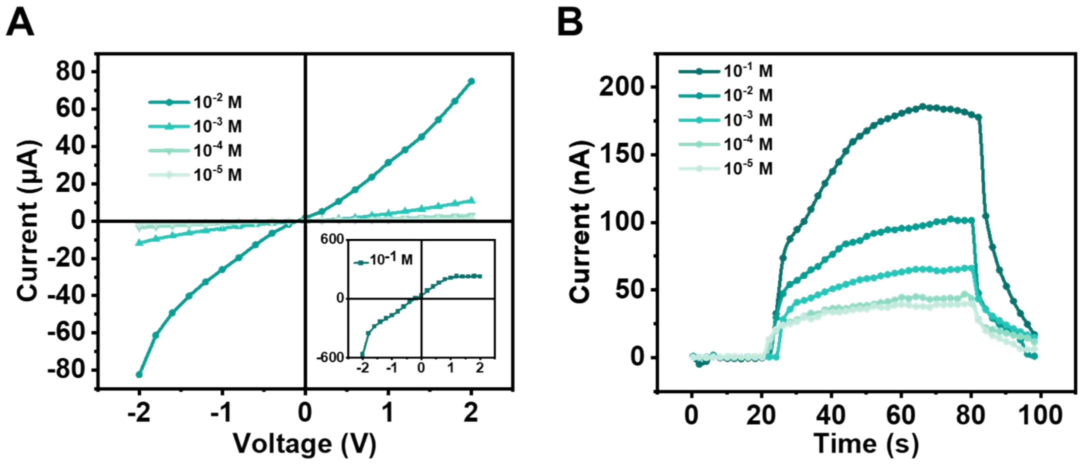

Figure S9. (A) Conventional external voltage-driven and (B) photothermal diffusion voltage-driven ion current responses MXene in KCl solutions with concentrations ranging from 1×10^{-1}M to 1×10^{-5} M.

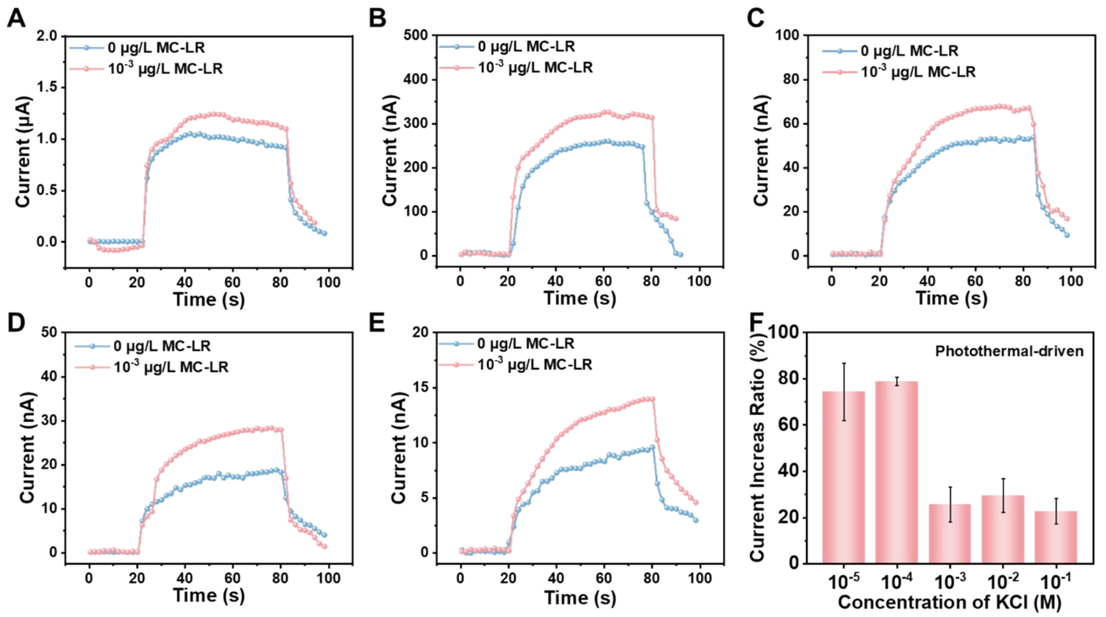

Figure S10 (A-E) Photothermal diffusion voltage-driven ion current responses of APT@MXene and APT@MXene + 10^{-3} μg/L MC-LR in KCl solutions with concentrations ranging from 1×10^{-1}M to 1×10^{-5} M. (F) Corresponding photothermal diffusion voltage-driven current increase ratio versus KCl concentration (1×10^{-1}M to 1×10^{-5} M).

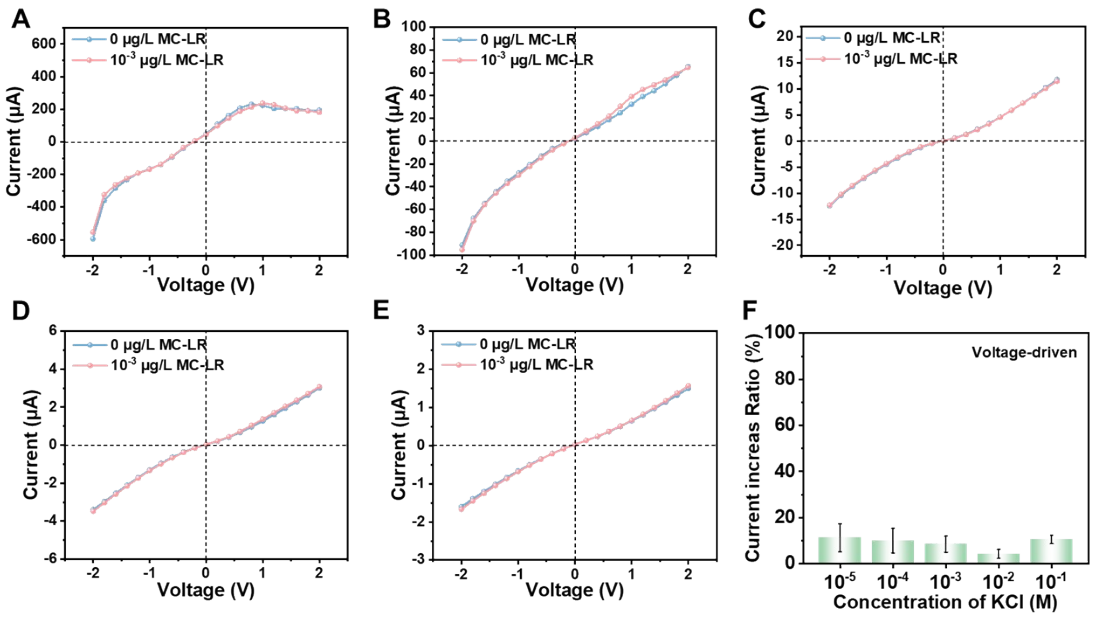

Figure S11 (A-E) Conventional external voltage-driven ion current responses of APT@MXene and APT@MXene + 10^{-3} μg/L MC-LR in KCl solutions with concentrations ranging from 1×10^{-1}M to 1×10^{-5} M. (F) Corresponding conventional external voltage-driven current increase ratio versus KCl concentration (1×10^{-1}M to 1×10^{-5} M).

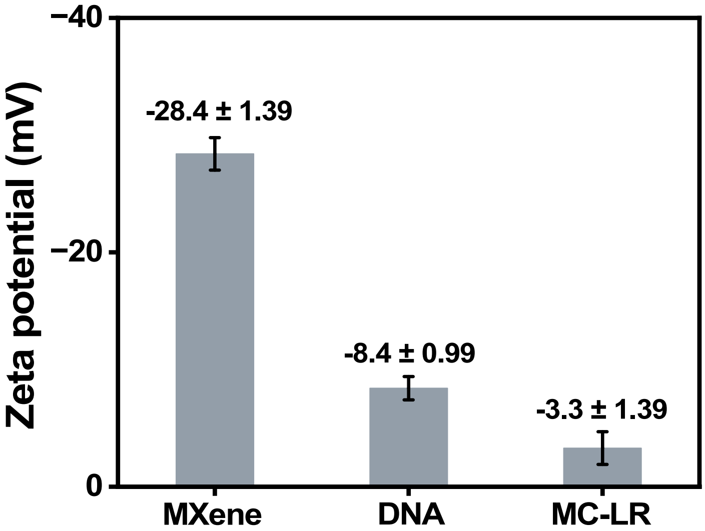

Figure S12 Zeta potential of MXen, DNA aptamer and MC-LR measured in 0.1 M KCl.

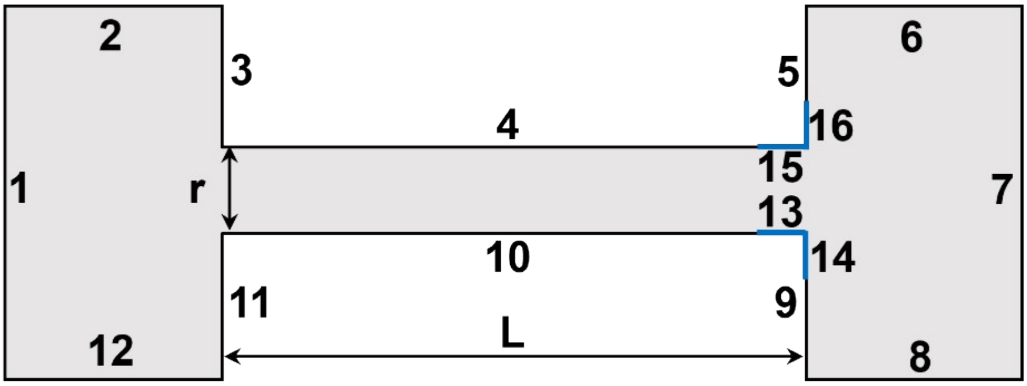

Figure S13 Scheme of numerical simulation model.

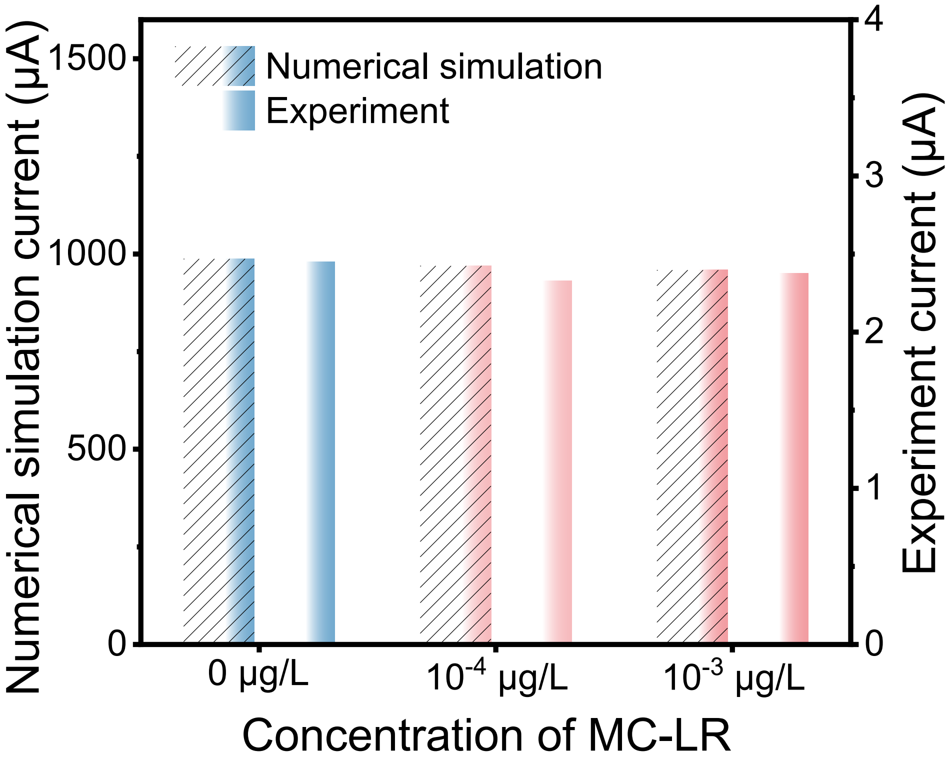

Figure S14 Simulated and experimental photothermal diffusion voltage-driven ion current for APT@MXene, APT@MXene + 10^{-4} μg/L MC-LR and APT@MXene + 10^{-3} μg/L MC-LR.

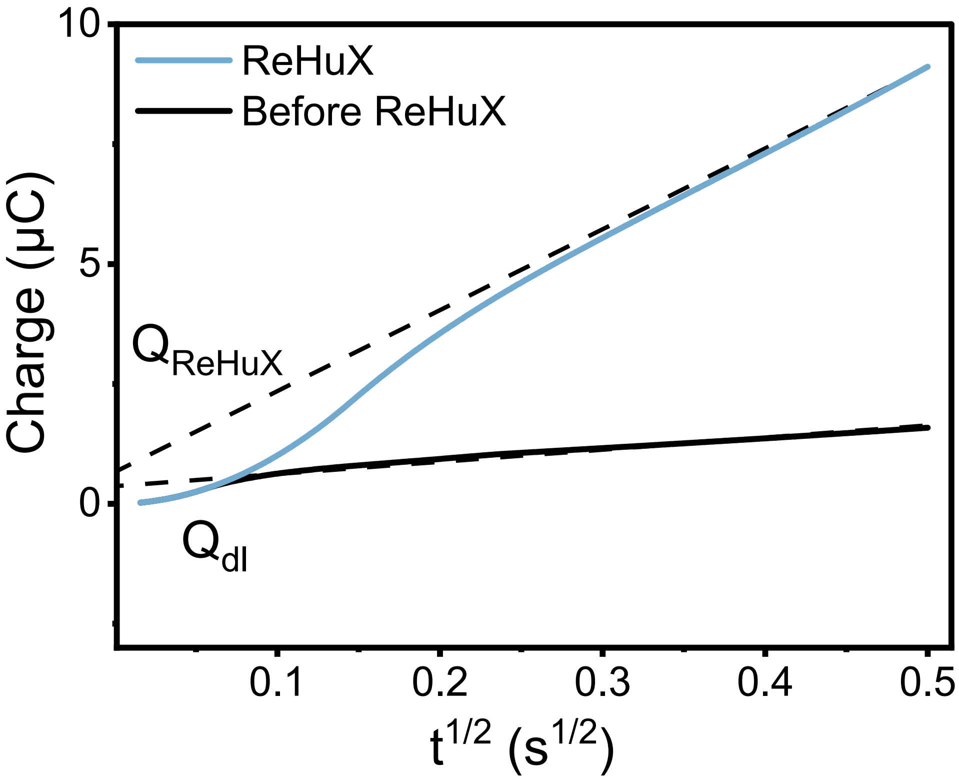

Figure S15 Chronocoulometry curves of nanochannels before and after the addition of RuHeX. Scan rate = 50 mV/s. Q_{dl} is obtained from the intercept at t = 0 in chronocoulometry curve in the absence of RuHeX, Q_{RuHeX} is obtained from the intercept at t = 0 in the presence of saturated RuHeX.

Supplementary tables

Table S1 The XPS date of MXene.

Peak	Binding energy (eV)	Atomic %
C1s	284.08	36.38
O1s	533.05	17.62
N1s	402.98	0
P2p	129.07	0
Ti2p	458.24	28.87

Table S2 The XPS date of APT@MXene.

Peak	Binding energy (eV)	Atomic %
C1s	284.92	49.88
O1s	531.22	29.27
N1s	398.98	13.61
P2p	132.75	7.25
Ti2p	459.08	0

Table S3 The XPS date of APT@MXene after Ar^+ aching.

Peak	Binding energy (eV)	Atomic %
C1s	284.25	28.14
O1s	532.86	25.78
N1s	401.74	0
P2p	136.16	0
Ti2p	457.24	32.74

Table S4 Comparison of currently reported methods for MC-LR detection

Method	Sample	Detection range (ng/mL)	LOD (ng/mL)	Year	Refs
$Ti_3C_2T_x$/MoS2/Au ECL immunosensor	Lake water	5×10^{-5} ~10	3.1×10^{-5}	2024	1
Aptameric photonic structure-based optical biosensor	Tape water	3.8~150	0.88	2024	2
Laser induced graphene electrochemical aptasensors	Lake water	1×10^{-3}~1×10^{2}	3×10^{-6}	2023	3
Thin-layer chromatography coupled with surface-enhanced Raman spectroscopy	Snail	5×10^{-3}~5×10^{2}	2.27×10^{-3}	2024	4
laser-induced graphene electrochemical biosensor	Agricultural irrigation water	5×10^{-3}~100	4.36×10^{-4}	2024	5
JNU-7-MIP based solid-phase extraction coupled with high performance liquid chromatography-mass spectrometry	Lake water<br>Soil	0.025~100	0.008	2024	6
CRISPR-Cas12a-based ECL biosensors	Lake water	0.01~50	1.2×10^{-3}	2024	7
Dual signal integrated sensing strategy of ECL and FL	Lake water	5×10^{-5}~200	1.1×10^{-5}	2023	8
Surface-Enhanced Raman	Lake water	0.01~100	0.014	2019	9

Scattering Immunosensor					
supersandwich DNA Probes modified aptasensors	Lake water	$1\times10^{-6}\sim1\times10^{2}$	2.5×10^{-7}	2024	10
This work	Tape water<br>Lake water	$1\times10^{-7}\sim1\times10^{-3}$	1×10^{-7}	2025	

Table S5 Boundary conditions for numerical simulation model.

Boundary	Poisson Equation	Nernst-Planck Equation
1	0 V	$c(K^+) = c(Cl^-) = c_0$
7	V_b V	$c(K^+) = c(Cl^-) = c_0$
2,3,5,6,8,9,11,12	Zero charge	$\vec{n} \cdot \vec{J}_i = 0$
4,10,13,14,15,16	$\vec{n} \cdot \nabla \varphi = -\frac{\sigma_S}{\varepsilon}$	$\vec{n} \cdot \vec{J}_i = 0$

References

1 Du, Y., Jiang, S., Yuan, H.-m., Cui, L. & Zhang, C.-y. Facile fabrication of silica nanoparticles with incorporation of AIEgen and coreactant as self-enhanced electrochemiluminescence probes for sensitive immunoassay of microcystin-LR. *Sensors and Actuators B: Chemical* **401**, doi:10.1016/j.snb.2023.134906 (2024).

2 Hussain, S., Al-Tabban, A. & Zourob, M. Aptameric photonic structure-based optical biosensor for the detection of microcystin. *Biosensors and Bioelectronics* **260**, doi:10.1016/j.bios.2024.116413 (2024).

3 Wang, Y. *et al.* Laser induced graphene electrochemical aptasensors based on tetrahedral DNA for ultrasensitive on-site detection of microcystin-LR. *Biosensors and Bioelectronics* **239**, doi:10.1016/j.bios.2023.115610 (2023).

4 Jiang, J., Liu, M., Xu, D., Jiang, T. & Zhang, J. Quantitative detection of microcystin-LR in Bellamya aeruginosa by thin-layer chromatography coupled with surface-enhanced Raman spectroscopy based on in-situ ZIF-67/Ag NPs/Au NWs composite substrate. *Food Chemistry* **452**, doi:10.1016/j.foodchem.2024.139481 (2024).

5 Chen, Z. *et al.* All in laser-induced graphene to fabricate the electrochemical biosensor for on-site detection of microcystin-LR. *Sensors and Actuators B: Chemical* **419**, doi:10.1016/j.snb.2024.136437 (2024).

6 Tang, Y.-H. *et al.* Engineering of molecularly imprinted cavity within 3D covalent organic frameworks: An innovation for enhanced extraction and removal of microcystins. *Journal of Hazardous Materials* **472**, doi:10.1016/j.jhazmat.2024.134469 (2024).

7 Shi, B. *et al.* Aggregation-Induced Electrochemiluminescence of Ir(ppy)3-Functionalized ZIF-8 for Microcystin-LR Detection via the trans-Cleavage Activity of CRISPR-Cas12a. *Analytical Chemistry* **96**, 15050-15058, doi:10.1021/acs.analchem.4c03495 (2024).

8 Zhao, Q. *et al.* Dual-Signal Integrated Aptasensor for Microcystin-LR Detection via In Situ Generation of Silver Nanoclusters Induced by Circular DNA Strand

Displacement Reactions. *Analytical Chemistry* **95**, 14317-14323, doi:10.1021/acs.analchem.3c02568 (2023).

9 Li, M., Paidi, S. K., Sakowski, E., Preheim, S. & Barman, I. Ultrasensitive Detection of Hepatotoxic Microcystin Production from Cyanobacteria Using Surface-Enhanced Raman Scattering Immunosensor. *ACS Sensors* **4**, 1203-1210, doi:10.1021/acssensors.8b01453 (2019).

10 Zhang, W. Q. *et al.* A Single Set of Well-Designed Aptamer Probes for Reliable On-site Qualitative and Ultra-Sensitive Quantitative Detection. *Angewandte Chemie International Edition* **63**, doi:10.1002/anie.202316434 (2024).
